# Correlation between *TCF7L2* and *CAPN10* gene polymorphisms and gestational diabetes mellitus in different geographical regions: a meta-analysis

**DOI:** 10.1186/s12884-023-06177-1

**Published:** 2024-01-02

**Authors:** Jingjing He, Meng Zhang, Jianhua Ren, Xiaolian Jiang

**Affiliations:** 1 Department of Obstetrics Nursing, West China Second University Hospital, Sichuan University, Chengdu, China; 2 West China School of Nursing, Sichuan University, Chengdu, China; 3 Key Laboratory of Birth Defects and Related Diseases of Women and Children (Sichuan University), Ministry of Education, Chengdu, Sichuan China

**Keywords:** TCF7L2, CAPN10, Gestational diabetes mellitus, Single nucleotide polymorphism, Meta-analysis

## Abstract

**Background:**

The association between *TCF7L2* and *CAPN10* gene polymorphisms and gestational diabetes mellitus (GDM) has been explored in diverse populations across different geographical regions. Yet, most of these studies have been confined to a limited number of loci, resulting in inconsistent findings. In this study, we conducted a comprehensive review of published literature to identify studies examining the relationship between *TCF7L2* and *CAPN10* gene polymorphisms and the incidence of GDM in various populations. We specifically focused on five loci that were extensively reported in a large number of publications and performed a meta-analysis.

**Methods:**

We prioritized the selection of SNPs with well-documented correlations established in existing literature on GDM. We searched eight Chinese and English databases: Cochrane, Elton B. Stephens. Company (EBSCO), Embase, Scopus, Web of Science, China National Knowledge Infrastructure (CNKI), Wanfang, and China Science and Technology Journal Database and retrieved all relevant articles published between the inception of the database and July 2022. The Newcastle Ottawa Scale (NOS) was used to evaluate the selected articles, and the odds ratio (OR) was used as the combined effect size index to determine the association between genotypes, alleles, and GDM using different genetic models. Heterogeneity between the studies was quantified and the *I*^*2*^ value calculated. Due to large heterogeneities between different ethnic groups, subgroup analysis was used to explore the correlation between genetic polymorphisms and the incidence of GDM in the different populations. The stability of the results was assessed using sensitivity analysis. Begg’s and Egger’s tests were used to assess publication bias.

**Results:**

A total of 39 articles reporting data on 8,795 cases and 16,290 controls were included in the analysis. The frequency of the rs7901695 genotype was statistically significant between cases and controls in the European population (OR = 0.72, 95% CI: 0.65–0.86) and the American population (OR = 0.61, 95% CI: 0.48–0.77). The frequencies of rs12255372, rs7901695, rs290487, and rs2975760 alleles were also considerably different between the cases and controls in the populations analyzed.

**Conclusions:**

rs7903146, rs12255372, rs7901695, rs290487, and rs2975760 were associated with the incidence of GDM in different populations.

## Background

Gestational diabetes mellitus (GDM) is defined as “glucose intolerance of different degrees that occurs or is discovered for the first time during pregnancy” [1]. It has a global incidence of approximately 14.0% [2]. The prevalence of GDM has increased by more than 30% in many countries over the past 30 years, while the birth rate has declined [3]. GDM affects both pregnant women and their offspring. GDM is a risk factor for gestational hypertension, gestational preeclampsia, and diabetes [4, 5]. Compared with the offspring of healthy women, the offspring of women with GDM are at considerably higher risk of developing obesity and diabetes in childhood and adulthood [6]; thus, GDM is an important public health concern [7]. However, the specific mechanism and factors influencing GDM remain unclear [8]. Genetic and environmental factors play important roles in the etiology of GDM [9]. Recent studies have found that single nucleotide polymorphisms (SNPs) in *TCF7L2, CAPN10, KCNQ1*, and *ADIPOQ* are associated with the onset of gestational diabetes [10, 11]. Of these, the earliest and most intensively studied are genes encoding transcription factor 7-like 2 (*TCF7L2*) and calpain 10 (*CAPN10*). The relationship between *TCF7L2* and *CAPN10* and the susceptibility to GDM has been studied in populations in different geographical regions. However, most of these studies have been based on a limited number of loci, and the results have been inconsistent. In this study, we screened published literature for studies on the relationship between *TCF7L2* and *CAPN10* gene polymorphisms and the incidence of diabetes mellitus during pregnancy in different populations. We selected five loci reported in a large number of articles and included them in a meta-analysis to determine genetic susceptibility to GDM in different populations.

## Methods

### Literature search

We prioritized the selection of SNPs with well-documented correlations established in existing literature on GDM. These specific SNPs, identified for their substantiated association with the onset or progression of GDM in previous research, provide a solid foundation of relevance to this condition. Additionally, our study involved an exhaustive review of published articles to pinpoint epidemiological studies exploring the relationship between SNPs within *TCF7L2* and *CAPN10* and their connection to gestational diabetes. This rigorous approach aimed to ensure a comprehensive understanding of the genetic variations associated with GDM and specifically targeted these genes for deeper investigation within the context of this condition. We searched eight Chinese and English databases: Cochrane, Elton B. Stephens. Company (EBSCO), Embase, Scopus, Web of Science, China National Knowledge Infrastructure (CNKI), Wanfang, and China Science and Technology Journal Database (VIP) using the search terms TCF7L2, transcription factor 7 like 2 protein, T-cell-specific transcription factor 4, T cell specific transcription factor 4, T cell transcription factor 4, TCF7L2 transcription factor, transcription factor, TCF7L2, or t cell factor 4 as well as monogenic diabetes, (diabetes, pregnancy induced), pregnancy-induced diabetes, gestational diabetes, (diabetes mellitus, gestational), or gestational diabetes mellitus. We also searched the Chinese databases CNKI, Wanfang, and Weipu using “gestational diabetes” or “GDM” and “transcription factor 7 analog-2” or “TCF7L2” as search terms. All relevant articles published between the date the database was established and July 2022 were retrieved. The articles were sorted by the name of the first author, the publication date, and ethnicity of study participants, and genotype and allele distribution information of the cases and controls were extracted.

### Inclusion criteria

The inclusion criteria were: (i) epidemiological studies on the correlation between *TCF7L2* SNPs and GDM; (ii) well-designed cohort, case–control, case-cohort, and cross-sectional studies; (iii) studies containing sufficient genotype or allele frequency data; and (iv) all cases included in the studies are patients diagnosed with GDM based on the GDM diagnostic criteria established by the American International Association of the Diabetes and Pregnancy Study Groups and "Obstetrics and Gynecology (Ninth Edition)."

### Exclusion criteria

The following publications were excluded: (i) abstracts, reviews, lectures, and dissertations; (ii) publications with incomplete or unavailable genotype or allele frequency data; (iii) studies reporting a combination of interventions; and (iv) studies reporting animal experiments.

### Data extraction

Three researchers independently screened original publications based on the inclusion criteria. Disagreements were resolved through discussion or by a fourth researcher. The following data were extracted from the publications that met the inclusion criteria: the name of the first author, the date of publication, definitions and characteristics of the case and control groups, and the distribution and frequency of alleles and genotypes in the included case and control groups.

### Evaluating the quality of publications

The Newcastle–Ottawa Scale (NOS) [12] was used to evaluate the quality of the screened publications. The final 39 articles included case and control groups. Articles with quality scores of 0–4 were classified as low-quality studies, while those with scores of 5–8 were classified as high-quality studies.

### Statistical analysis

Stata16.0 software was used to calculate odds ratios (OR) to represent the combined effect size index. Correlations between genotypes, alleles, and GDM were analyzed using different models. Heterogeneity between studies was tested before merging the results. Where there was large heterogeneity between studies, subgroup analysis was used to explore the source of heterogeneity. Heterogeneity between studies was quantitatively analyzed and the *I*^*2*^ value calculated. Where* p* < 0.1 or* I*^*2*^ > 40%, a random effect model was selected to calculate the combined OR value and the 95% confidence interval (Cl), otherwise, a fixed effect model was used. To evaluate the sensitivity of the analyses and to test the stability and reliability of the meta-analysis results, individual studies were excluded in turn and the combined OR and 95% Cl were recalculated. To avoid false positives due to multiple comparisons, pooled effect size values were corrected using the false discovery rate. Begg's and Egger's tests were used to determine publication bias [13]. The test level was set at* α* = 0.05.

## Results

### Description of the included studies

**Fig. 1:**
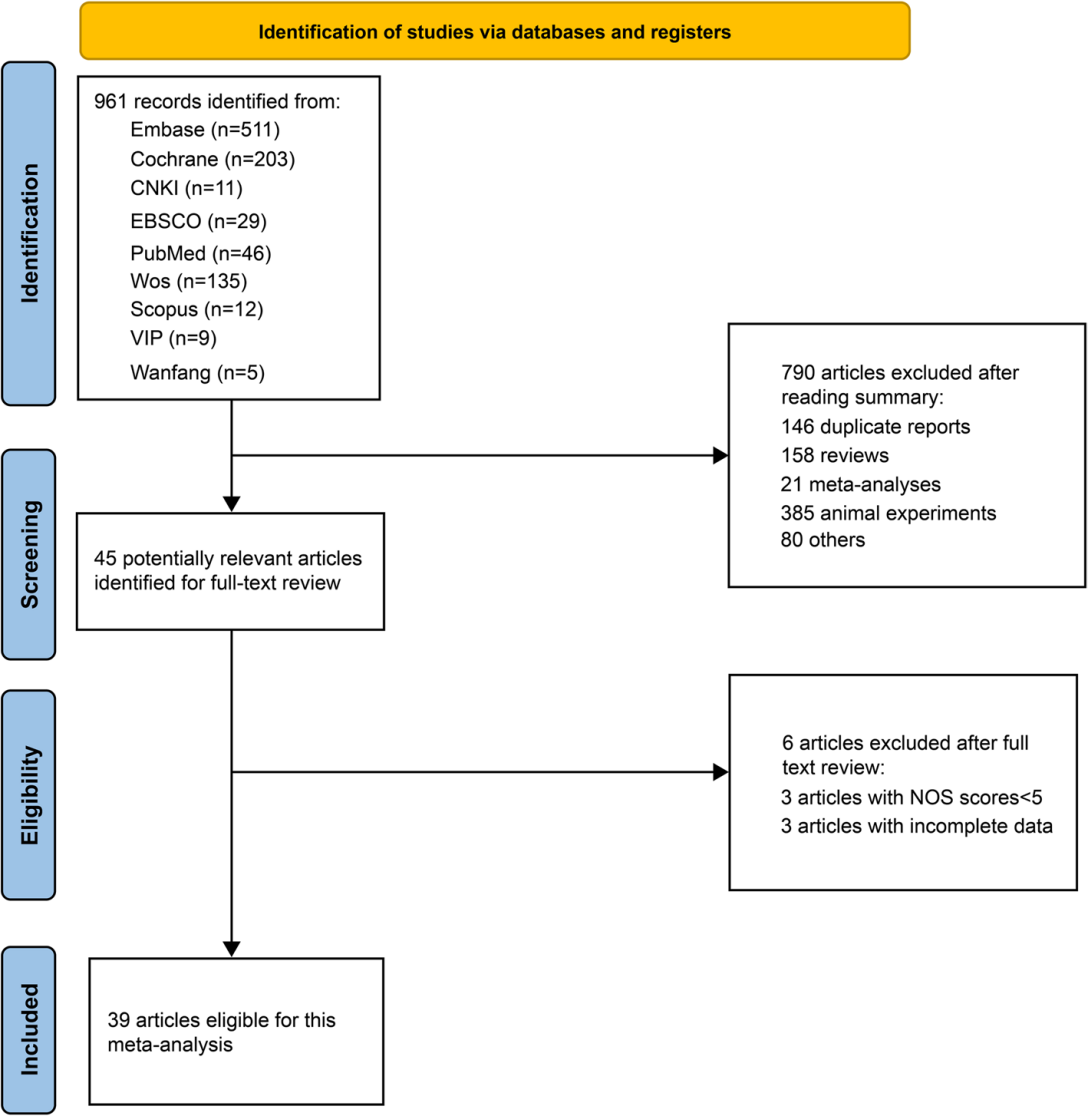
PRISMA flowchart

A total of 25 publications written in Chinese and 936 publications written in English were retrieved using the specified search terms. Duplicated publications were removed, leaving 16 articles written in Chinese and 799 articles written in English. Based on the exclusion and inclusion criteria, 644 articles were excluded after reading the abstracts. Three articles with NOS scores < 5 and three articles with incomplete data were excluded after reading the full text. A total of 39 articles reporting data on 8,795 cases and 16,290 controls were included in the final analysis. The process and outcome of database screening are shown in Fig. 1. Basic information on each included article is given in Table 1, while case data is provided in Table 2.

**Table 1:** Characteristics of publications included in this study

Publication	Region	Country	Mean age(cases/controls)	Genotyping method	GDM criteria	Newcastle Ottawa Scale (NOS)
Popova 2021 [14]	Europe	Russia	31.9/29.5	SNP Genotyping Assay	OGTT	7
Gorczyca 2016 [15]	Europe	Poland	30.36/30.88	Sequencing	OGTT	7
Fd 2020 [16]	Europe	Lithuania	53.1/60.2	TaqMan allelic discrimination assay	WHO	8
Freathy 2010 [17]	Europe/Pacific	UK and Australia	NA	illumina Golden Gate platform	OGTT	6
Lauenborg 2009 [18]	Europe	Denmark	43.1/42.5	TaqMan allelic discrimination assay	OGTT	6
Pagan 2014 [19]	Europe	Spain	34.31/31.2	Sequencing	OGTT	6
Papadopoulou 2011 [20]	Europe	Sweden	NA	TaqMan allelic discrimination assay	OGTT	6
Pappa 2011 [21]	Europe	Germany	32.5/26.67	PCR	OGTT	7
Shaat 2007 [22]	Europe	Scandinavia	32.3/30.5	TaqMan allelic discrimination assay	OGTT	6
Včelák 2012 [23]	Europe	Czech Republic	32.8/29.9	TaqMan allelic discrimination assay	WHO	7
Shalabi 2021 [14]	Africa	Egypt	27.9/27.2	PCR	OGTT	8
de Melo 2015 [24]	America	Brazil	32/24	SNP Genotyping Assay	ADA	7
Huerta 2015 [25]	America	Mexico	35/28	SNP genotype (LGC)	OGTT	6
Reyes 2014 [26]	America	Mexico	29/31	PCR	ADA	6
Yadav 2016 [27]	Asia	India	28.12/30.65	PCR	OGTT	7
Li 2016 [28]	Asia	China	30.06/28.67	PCR	OGTT	7
Chen 2019 [29]	Asia	China	29.9/http: / /grch37. ensembl. org /index. Html	PCR	OGTT	8
Chen 2019 [30]	Asia	China	28.7/29.5	PCR	OGTT	8
Aris 2011 [31]	Asia	Malaysia	29.7/28.5	NA	ADA	6
Cho 2009 [32]	Asia	Korea	32/64.7	Allelic discrimination assay	IWCGDM	6
Kan 2014 [33]	Asia	China	30.7/30.9	Allecic discrimination assay	OGTT	6
RIZK 2011 [34]	Asia	Qatar	NA	TaqMan allelic discrimination assay	OGTT	6
Shi 2014 [35]	Asia	China	30/29	AS-PCR	OGTT	7
Thomas 2014 [36]	Asia	India	NA	NA	NA	6
Zhang 2015 [37]	Asia	China	30.58/28.75	PCR-LDR	OGTT	7
Klein 2012 [38]	Pacific	Australia	28.2/30.1	NA	OGTT	6
Isfer 2017 [39]	America	Brazil	30.6/31.9	PCR	ADA	8
Stuebe 2014 [40]	America	USA	24.1(toal)	Sequenom Iplex platform	OGTT	7
Li 2016 [41]	Asia	China	35.50/28.81	PCR	OGTT	8
Watanabe 2007 [42]	America	USA	30.58/28.75	PCR–RFLP	OGTT	6
Ye 2016 [43]	Asia	China	28.9/29.2	SNP genotype (LGC)	OGTT	7
Hui 2011 [44]	Asia	China	32/30	PCR-LDR	OGTT	6
Liu 2014 [45]	Asia	China	32.5/29.9	PCR	OGTT	6
Imran 2014 [46]	Asia	India	26.7/24	PCR	OGTT	6
Shaat 2005 [47]	Europe	Sweden	32.2/30.5	SNP genotype (LGC)	OGTT	5
Anna 2018 [48]	America	Mexico	32.13/30.63	TaqMan qPCR assay	OGTT	8
Ustianowski 2021 [49]	Europe	Poland	30.3/31.7	PCR	OGTT	8
Zhang 2019 [50]	Asia	China	28.2/27.5	PCR	OGTT	8

*SNP* single nucleotide polymorphism, *Qpcr* quantitative polymerase chain reaction

**Table 2:** *TCF7L2* and *CAPN10* allele distributions in gestational diabetes mellitus cases and controls

Variant (minor allele)	Author	Region	Genotypes in GDM cases				Genotypes in GDM controls				P for HWE
			n	CC	CT	TT	n	CC	CT	TT	
rs7903146	Popova	Europe	684	42	255	387	449	27	154	268	*p* > 0.05
	Gorczyca-Siudak	Europe	50	19	29	2	26	10	15	1	*p* > 0.05
	Fd	Europe	158	87	68	3	300	203	89	8	*p* < 0.05
	Lauenborg	Europe	276	33	125	118	2353	198	863	1292	*P* < 0.05
	Pagan	Europe	45	8	18	19	24	2	12	10	*p* > 0.05
	Papadopoulou	Europe	803	88	352	363	1110	82	384	644	*P* < 0.05
	Shaat	Europe	585	59	255	271	1111	69	392	650	*P* < 0.05
	Shalabi	Africa	114	18	53	43	114	41	62	11	*P* < 0.05
	de Melo	America	200	20	104	76	200	16	86	98	*P* < 0.05
	Huerta-Chagoya	America	408	19	124	265	342	10	67	265	*P* < 0.05
	Reyes-Lopez	America	90	6	29	55	108	4	23	81	*p* > 0.05
	Yadav	Asia	102	102	0	0	487	484	3	0	NA
	Li	Asia	100	92	8	0	100	96	4	0	NA
	Chen	Asia	98	29	31	38	120	9	47	64	*P* < 0.05
	Chen	Asia	155	144	11	0	159	150	9	0	NA
	Aris	Asia	173	129	43	1	114	99	15	0	*P* < 0.05
	Chao	Asia	868	2	63	803	627	0	31	596	*p* > 0.05
	Freathy	Asia	384	0	46	338	1322	3	108	1211	NA
	Kan	Asia	100	1	15	84	100	0	5	95	*P* < 0.05
	RIZK	Asia	40	6	18	16	74	8	37	29	*p* > 0.05
	Shi	Asia	100	24	36	40	100	7	38	55	*P* < 0.05
	Thomas	Asia	117	16	46	55	49	4	18	27	*p* > 0.05
	Zhang	Asia	113	0	17	96	115	0	5	110	NA
	Klein	Pacific	125	5	112	8	123	8	8	107	*P* < 0.05
	Freathy	Europe	614	75	246	293	3811	370	1557	1884	*P* < 0.05
	Pappa	Europe	148	18	81	49	107	7	38	62	*P* < 0.05
rs12255372	Taghreed	Africa	114	31	55	28	114	60	45	9	*P* < 0.05
	Li	Asia	100	98	2	0	100	100	0	0	NA
	Li	Asia	30	30	0	0	32	32	0	0	NA
	Cho	Asia	867	0	7	860	630	0	2	628	NA
	Nasser	Asia	40	6	28	6	74	11	38	25	*P* < 0.05
	Shi	Asia	100	0	0	100	100	0	0	100	NA
	Klein	Pacific	125	125			125	125			NA
	Polina	Europe	295	21	93	181	191	11	61	119	*p* > 0.05
	Migle	Europe	157	89	64	4	300	202	89	9	*P* < 0.05
	Pagan	Europe	45	6	20	19	25	2	14	9	*p* > 0.05
	Papadopoulou	Europe	794	95	356	343	1102	90	405	607	*P* < 0.05
	Vcelak	Europe	261	17	102	142	376	35	185	156	*P* < 0.05
	Reyes-Lopez	America	90	7	23	60	108	2	5	101	*P* < 0.05
	Watanabe	America	94	94			58	58			NA
	Watanabe	America	200	20	88	92	200	23	75	102	*p* > 0.05
rs7901695	Michalak-Wojnowska	Europe	50	19	30	1	26	9	16	1	*P* < 0.05
	Fd	Europe	158	3	76	79	299	12	99	188	*P* < 0.05
	Pagan	Europe	45	8	20	17	25	2	13	10	*p* > 0.05
	Papadopoulou	Europe	794	95	356	343	1102	90	405	607	*P* < 0.05
	Vcelak	Europe	261	25	130	106	376	24	147	205	*P* < 0.05
	Stuebe	America	56	9	30	17	842	70	357	415	*P* < 0.05
	Isfer	America	127	44	67	16	125	52	62	11	*p* > 0.05
	Stuebe	Africa	24	4	15	5	362	79	162	121	*p* > 0.05
rs290487	Ye	Asia	556	110	223	223	496	21	235	240	*P* < 0.05
	Jia	Asia	155	25	69	61	159	18	88	53	*p* > 0.05
	Hui	Asia	480	90	220	170	631	88	282	261	*P* < 0.05
	Shi	Asia	100	12	36	52	90	6	34	50	*p* > 0.05
	Liu	Asia	70	11	37	22	70	9	33	28	*p* > 0.05
SNP-44 rs2975760	Imran	Asia	137	85	40	12	150	97	42	11	*p* > 0.05
	Shaat	Europe	226	32	177	17	1181	787	351	43	*p* > 0.05
	Anna	America	116	93	22	1	83	64	18	1	*P* < 0.05
	Ustianowski	Europe	270	196	67	7	347	261	77	9	*P* < 0.05
	Zhang	Asia	138	53	68	17	152	65	79	8	*P* < 0.05

*GDM* gestational diabetes mellitus, *HWE* Hardy–Weinberg equilibrium

### Meta-analysis results

#### Association between rs7903146 and GDM

A total of 26 articles reporting data on 6,650 cases and 13,545 controls were included in this analysis. rs7903146 was the most common SNP in *TCF7L2*. Analysis using five gene models showed that polymorphisms in this locus were associated with the incidence of GDM in African, Asian, American, and Pacific populations.

**Fig. 2:**
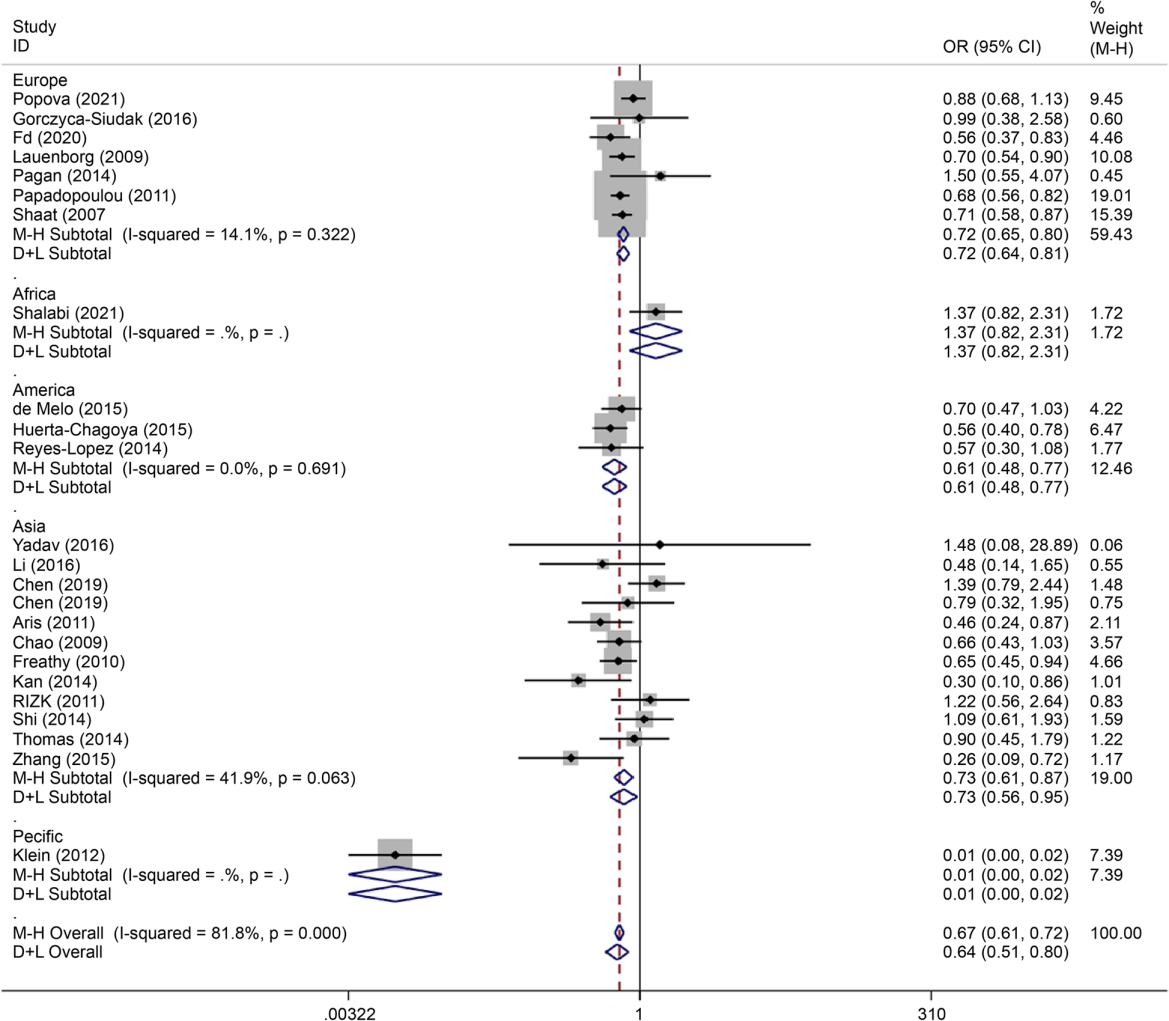
Forest plot of the relationship between GDM and rs 7,903,146 SNP under overdominance model. GDM, gestational diabetes mellitus; SNP, single nucleotide polymorphisms. Black diamonds denote the odds ratio

Over-dominant gene model (CC + TT/CT) analysis showed very high heterogeneity (*I*^*2*^ = 81.8% *P* < *0.0001*). Subgroup analysis conducted to reduce heterogeneity showed that rs7903146 was associated with the incidence of GDM in European (OR = 0.72, 95% CI: 0.65–0.86), American (OR = 0.61, 95% CI: 0.48–0.77), Asian (OR = 0.61, 95% CI: 0.48–0.77), African (0.73, 95% CI: 0.61–0.87), and Pacific (OR = 0.01, 95% CI: 0.00–0.02) populations (Fig. 2).

**Fig. 3:**
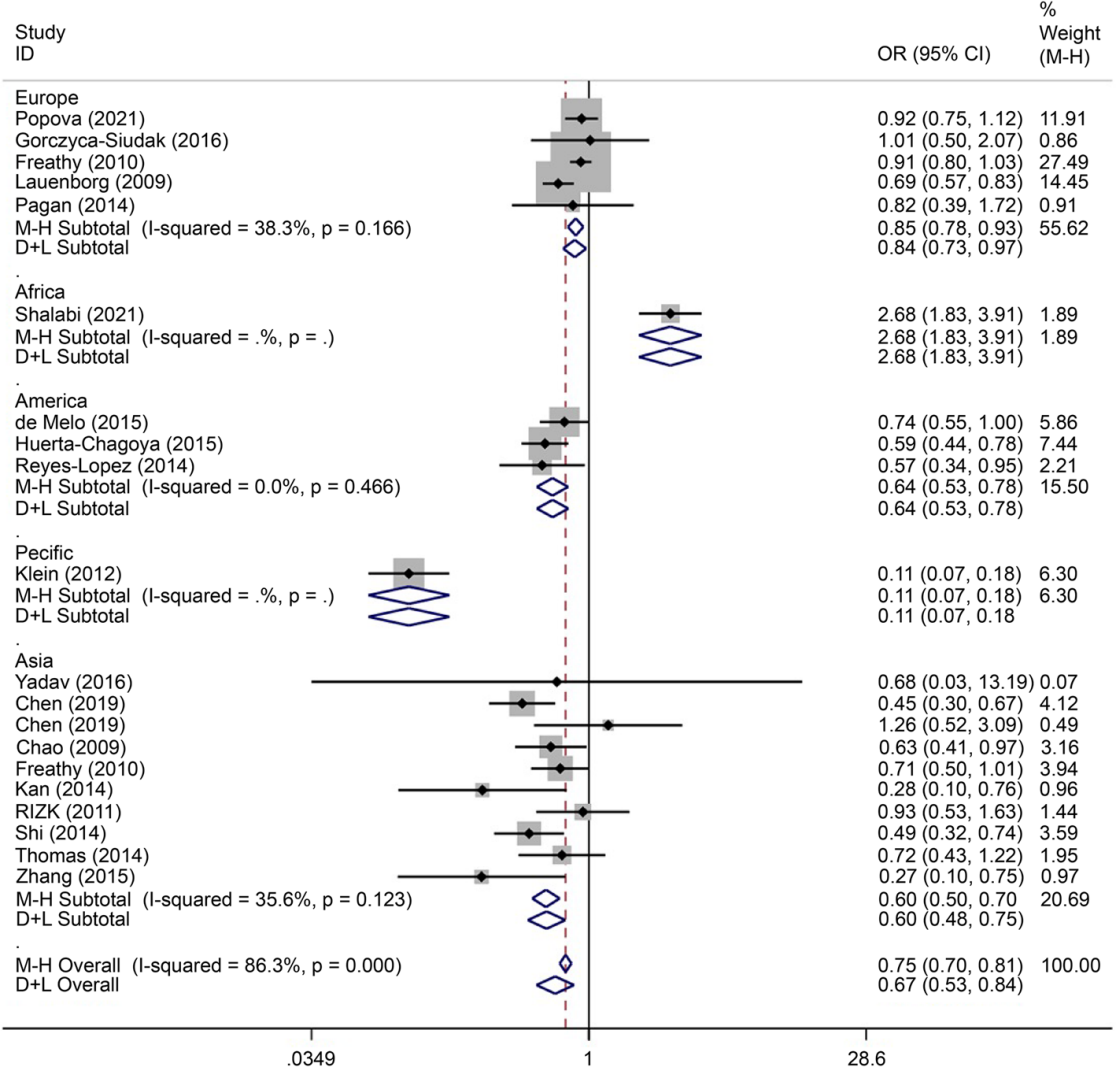
Forest plot of the relationship between GDM and rs 7,903,146 SNP under allelic model. GDM, gestational diabetes mellitus; SNP, single nucleotide polymorphisms. Black diamonds denote the odds ratio

Allelic model (T/C) subgroup analysis identified more mutations in this locus in the European (OR = 0.85, 95% CI: 0.78–0.9), African (OR = 2.68, 95% CI: 1.83–3.91), American (OR = 0.64, 95% CI: 0.53–0.78), Asian (OR = 0.60, 95% CI: 0.50–0.70), and Pacific (OR = 0.11, 95% CI: 0.07–0.18) populations (Fig. 3). The rs7903146 SNP variant, which is a risk factor for people from America, Asia, and the Pacific, may be a protective factor for Africa.

**Fig. 4:**
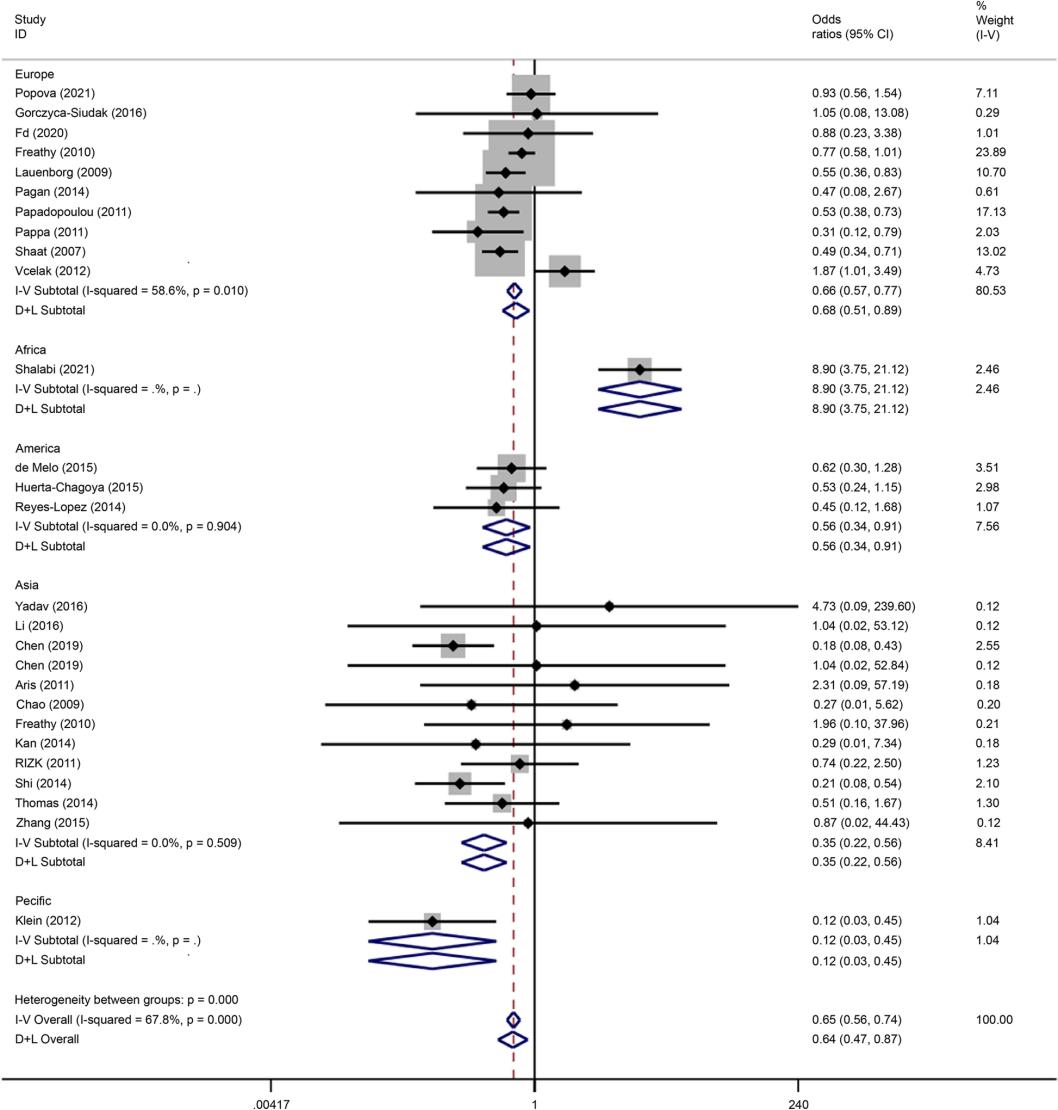
Forest plot of the relationship between GDM and rs 7,903,146 SNP under codominant model. GDM, gestational diabetes mellitus; SNP, single nucleotide polymorphisms. Black diamonds denote the odds ratio

**Fig. 5:**
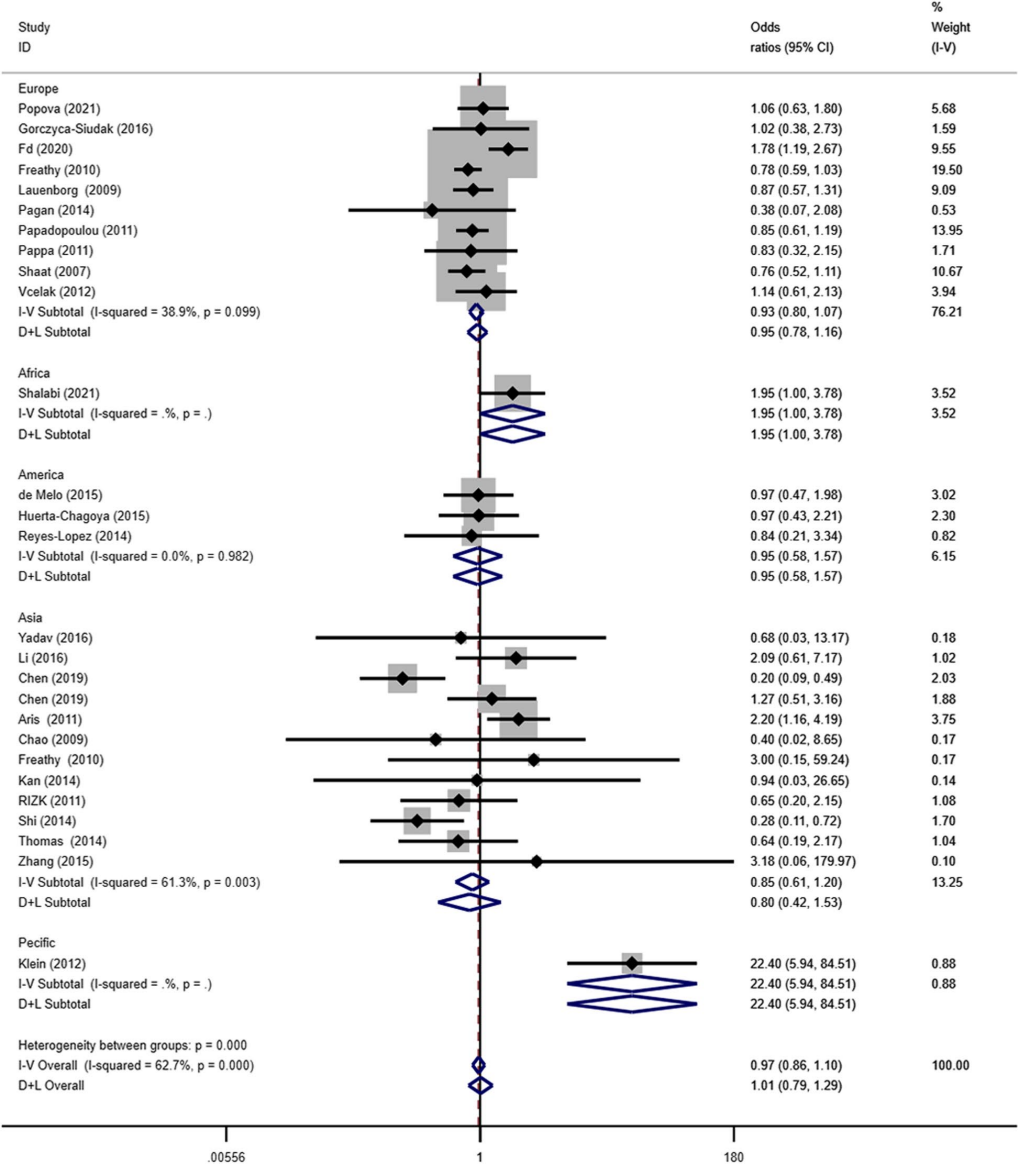
Forest plot of the relationship between GDM and rs 7,903,146 SNP under codominant model 2. GDM, gestational diabetes mellitus; SNP, single nucleotide polymorphisms. Black diamonds denote the odds ratio

Subgroup analysis based on the co-dominant gene model (TT/CC) showed that the polymorphism was associated with GDM pathogenesis in the European (OR = 0.66, 95% CI: 0.57–0.77), African (OR = 8.90, 95% CI: 3.75–21.12), American (OR = 0.56, 95% CI: 0.34–0.97), Asian (OR = 0.35, 95% CI: 0.22–0.56), and Pacific (OR = 0.12, 95% CI: 0.03–0.45) populations (Fig. 4). The co-dominant gene model (CT/CC) subgroup analysis showed that this polymorphism was associated with the incidence of GDM in the Pacific or Asian populations (OR = 22.45, 95% CI: 5.94–84.51), as shown in Fig. 5.

**Fig. 6:**
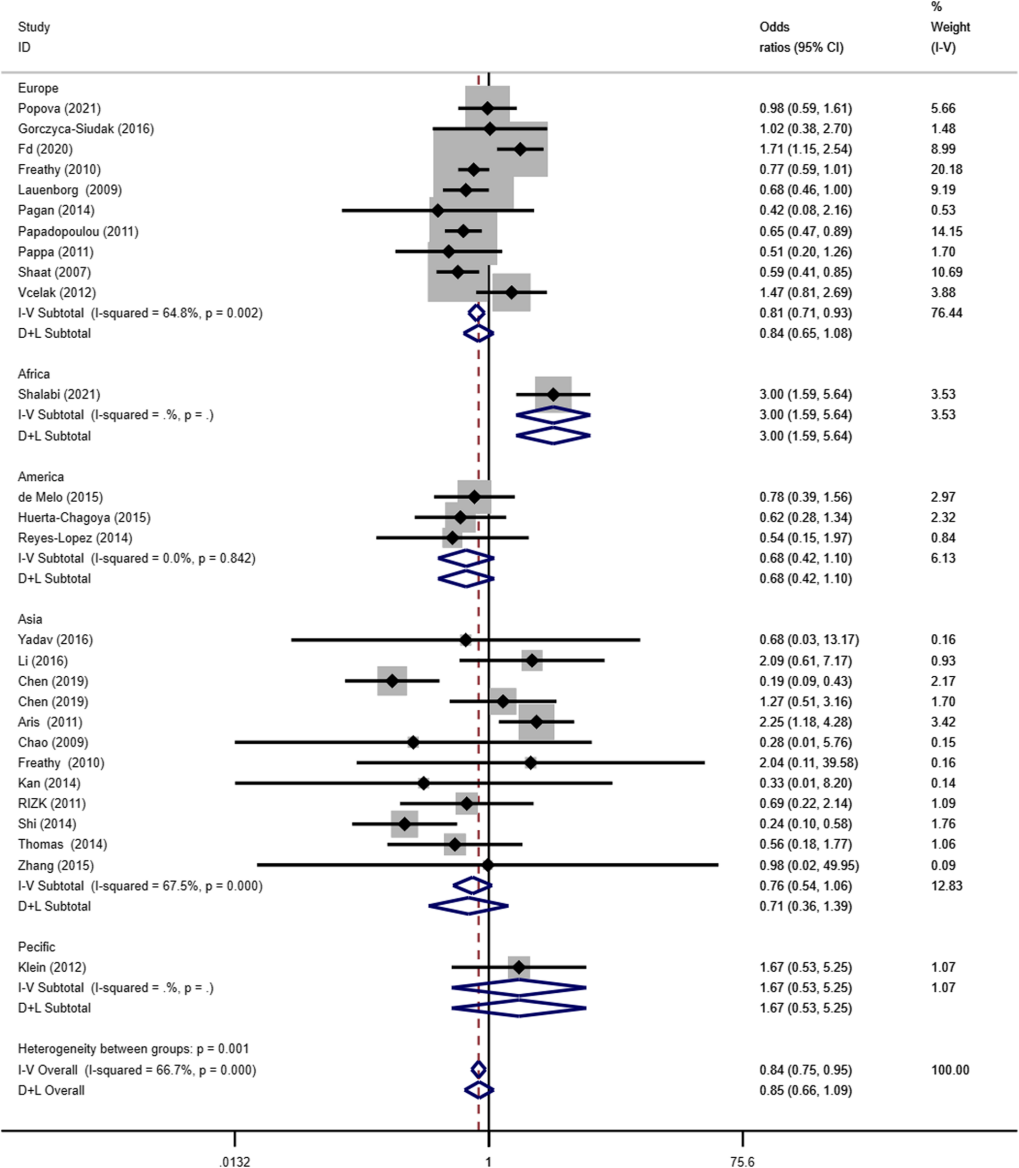
Forest plot of the relationship between GDM and rs 7,903,146 SNP under dominant model. GDM, gestational diabetes mellitus; SNP, single nucleotide polymorphisms. Black diamonds denote the odds ratio

The dominant gene model (CT + TT/CC) subgroup analysis suggested that the SNP was associated with GDM pathogenesis in the European (OR = 0.81, 95% CI: 0.71–0.93 =) and African (OR = 3.00, 95% CI: 1.59–5.64) populations (Fig. 6).

**Fig. 7:**
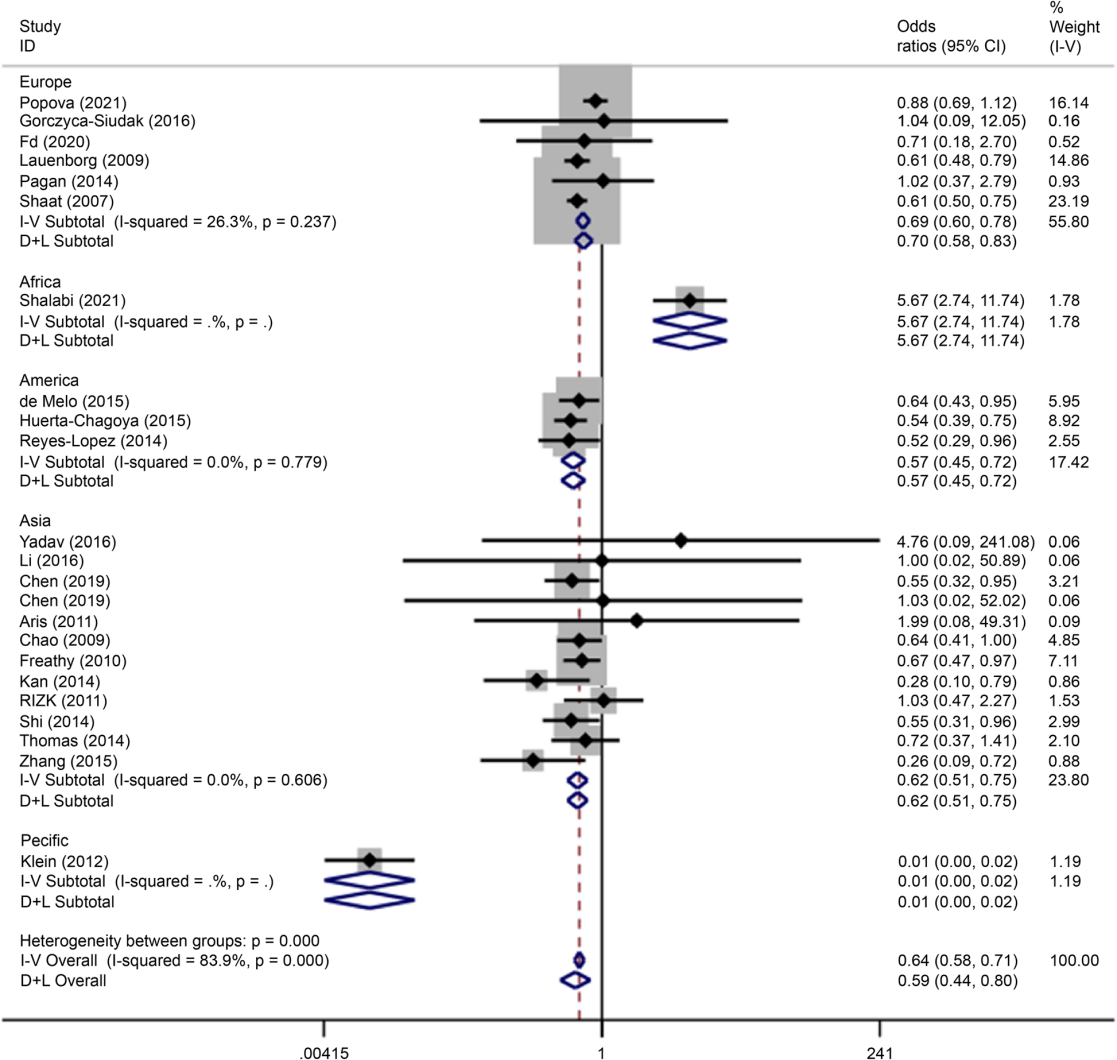
Forest plot of the relationship between GDM and rs 7,903,146 SNP under recessive model. GDM, gestational diabetes mellitus; SNP, single nucleotide polymorphisms. Black diamonds denote the odds ratio

Recessive gene model (TT/CC + CT) analysis showed that the polymorphism was associated with the onset of GDM in the European (OR = 0.69, 95% CI: 0.60–0.78), African (OR = 5.67, 95% CI: 2.74–11.74), American (OR = 0.57, 95% CI: 0.45–0.72), Asian (OR = 0.62, 95% CI: 0.51–0.75), and Pacific (OR = 0.01, 95% CI: 0.00–0.02) populations (Fig. 7).

#### Association between rs12255372 and GDM

**Fig. 8:**
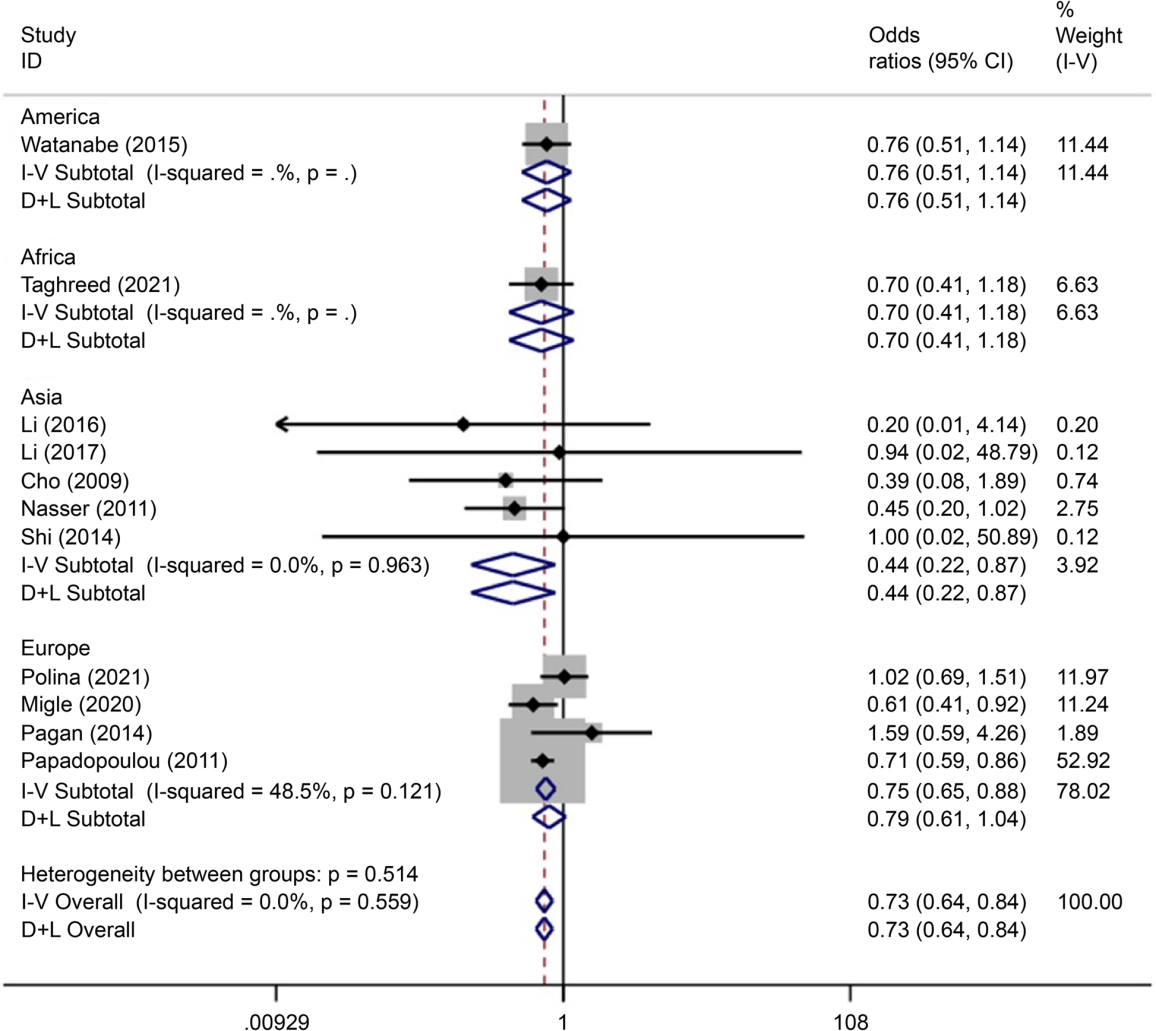
Forest plot of the relationship between GDM and rs 12,255,372 SNP under over-dominant model. GDM, gestational diabetes mellitus; SNP, single nucleotide polymorphisms. Black diamonds denote the odds ratio

**Fig. 9:**
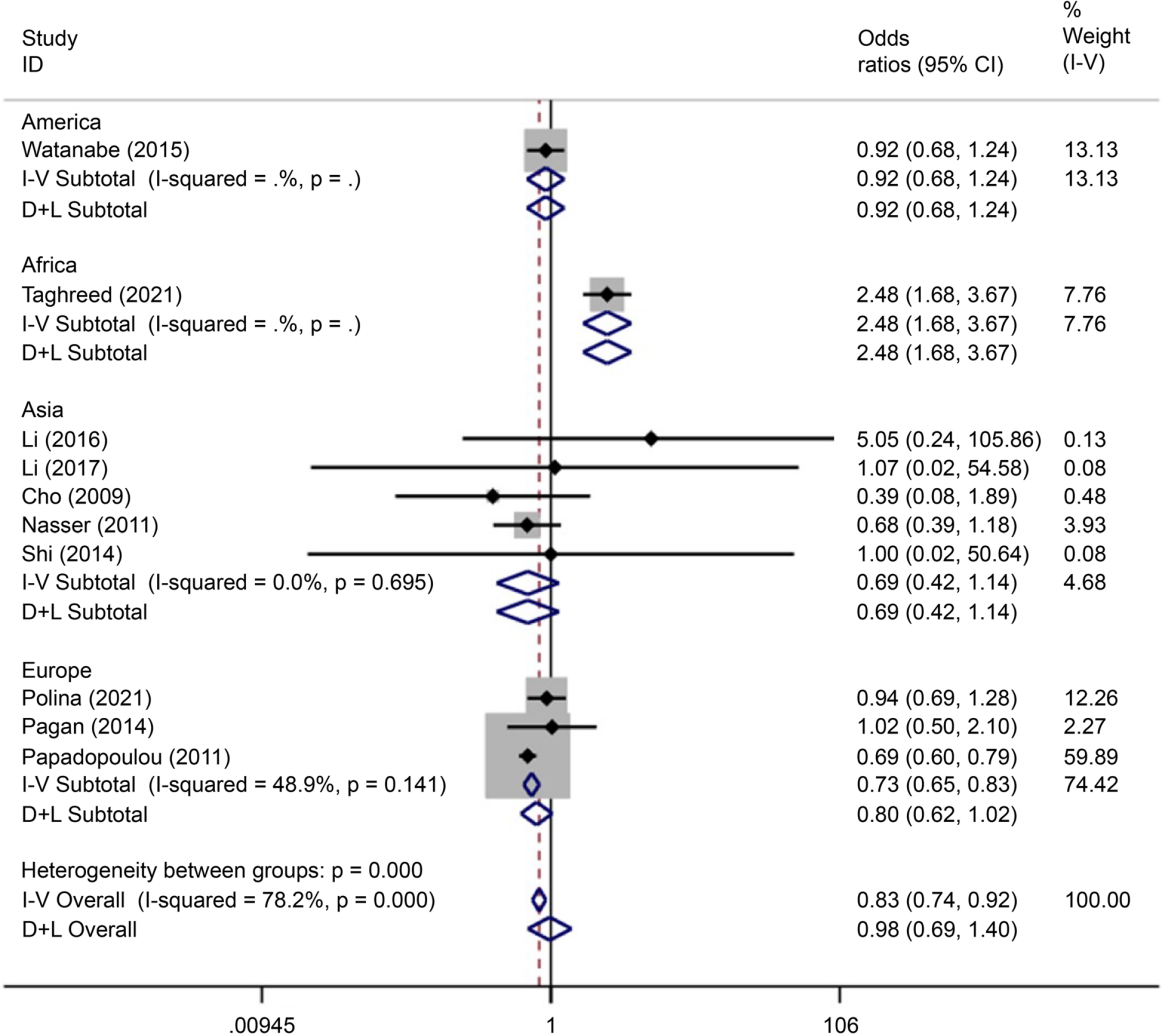
Forest plot of the relationship between GDM and rs 12,255,372 SNP under allelic model. GDM, gestational diabetes mellitus; SNP, single nucleotide polymorphisms. Black diamonds denote the odds ratio

**Fig. 10:**
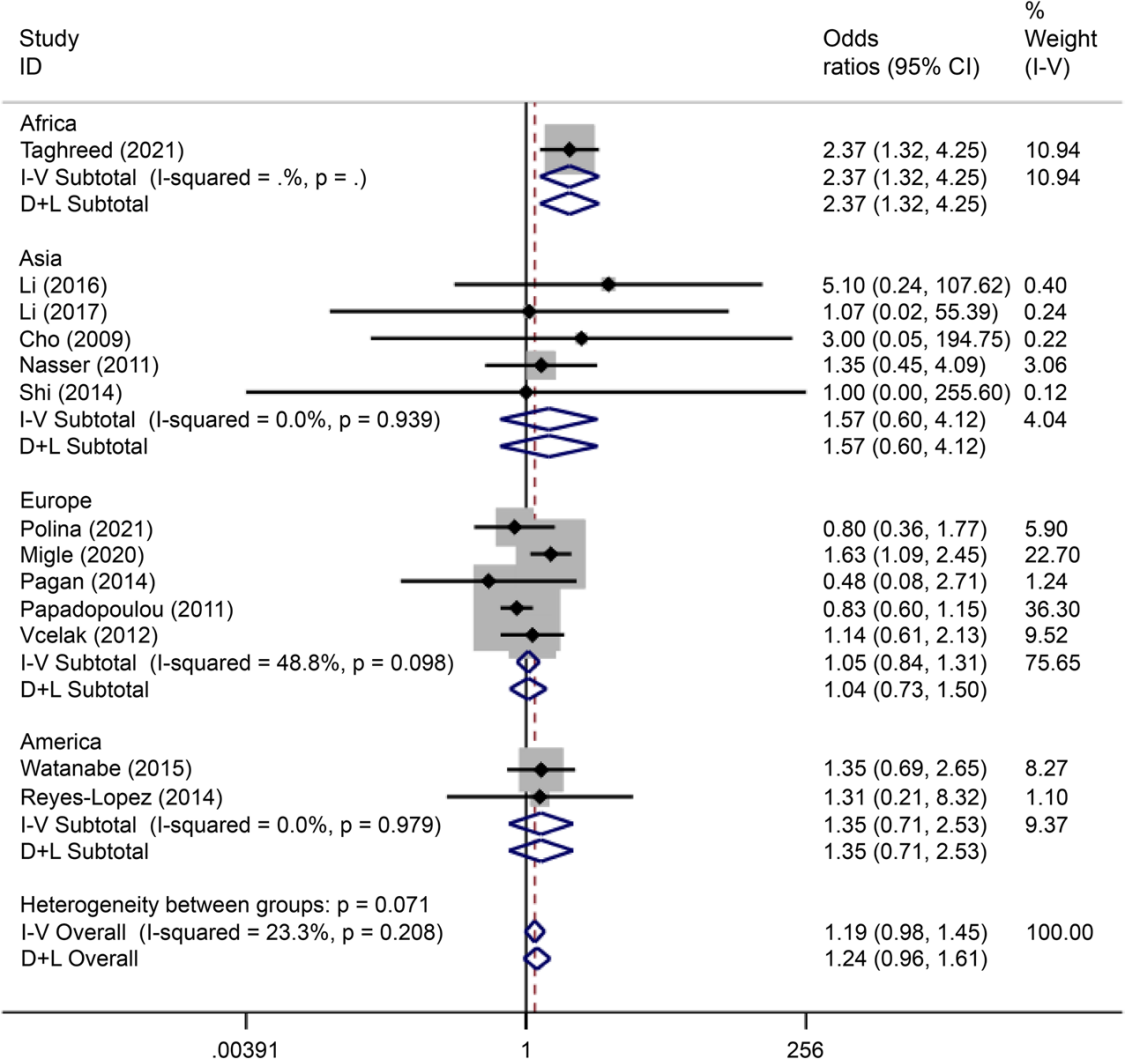
Forest plot of the relationship between GDM and rs 12,255,372 SNP under codominant model 2. GDM, gestational diabetes mellitus; SNP, single nucleotide polymorphisms. Black diamonds denote the odds ratio

**Fig. 11:**
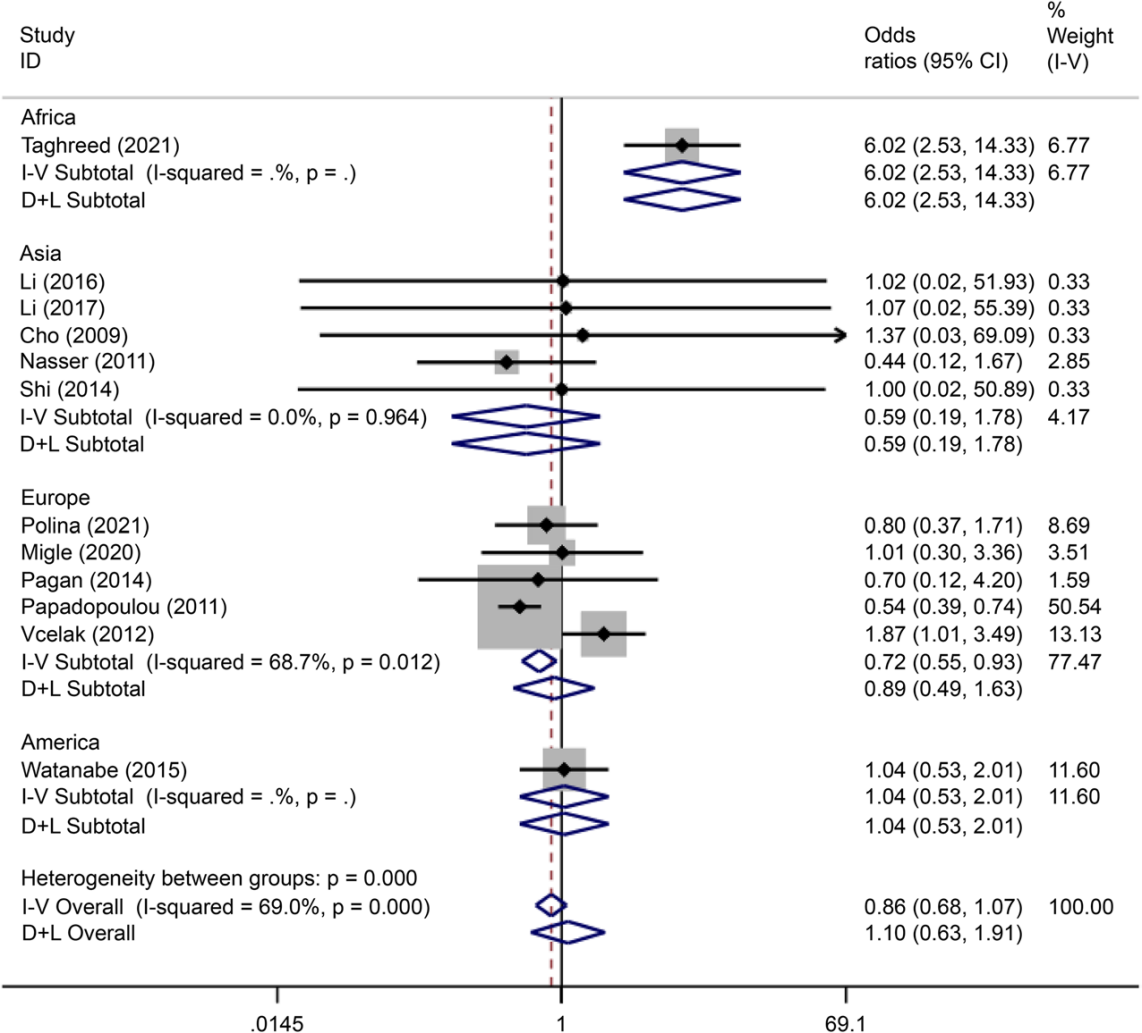
Forest plot of the relationship between GDM and rs 12,255,372 SNP under codominant model 1. GDM, gestational diabetes mellitus; SNP, single nucleotide polymorphisms. Black diamonds denote the odds ratio

**Fig. 12:**
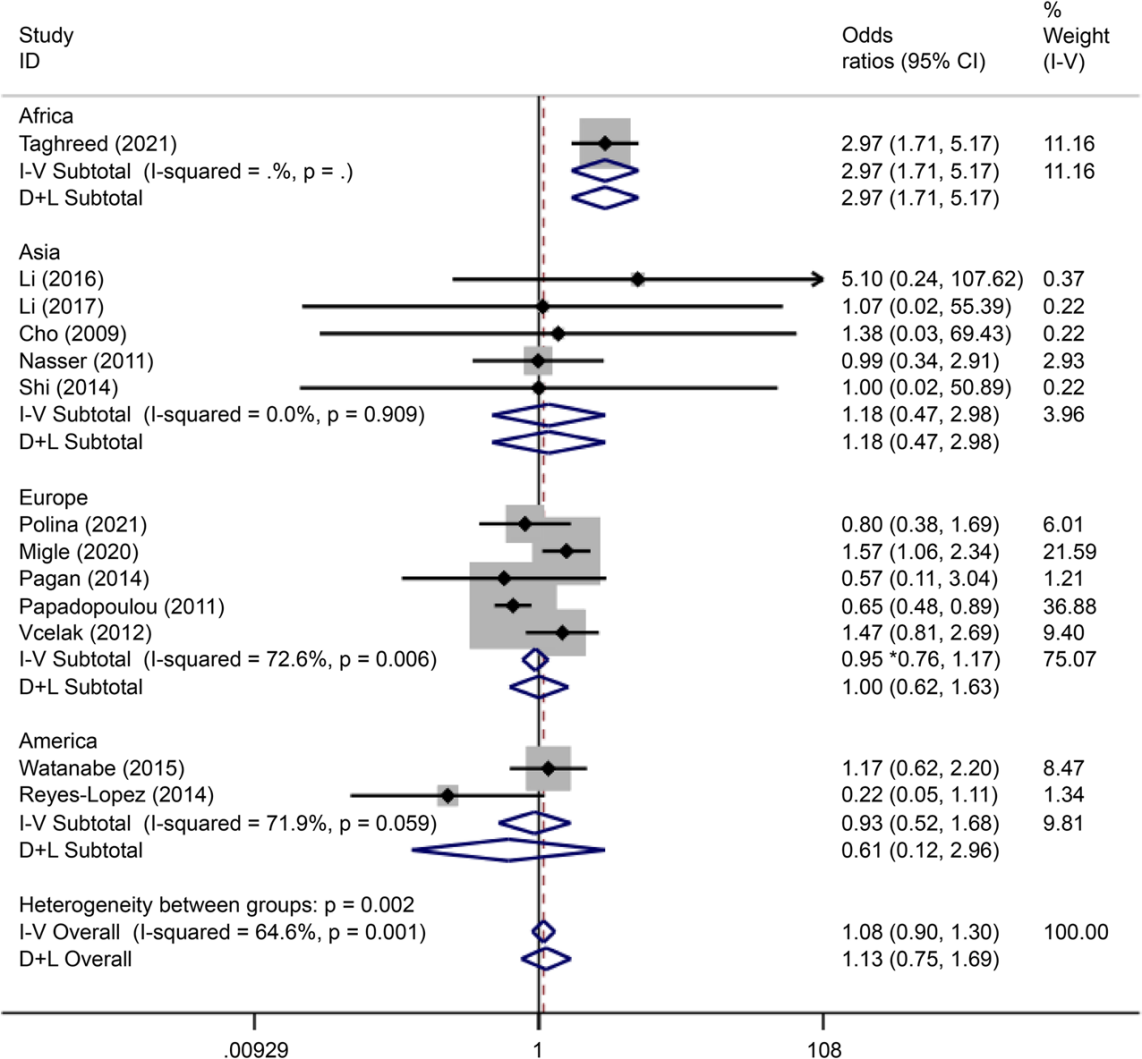
Forest plot of the relationship between GDM and rs 12,255,372 SNP under dominant model. GDM, gestational diabetes mellitus; SNP, single nucleotide polymorphisms. Black diamonds denote the odds ratio

**Fig. 13:**
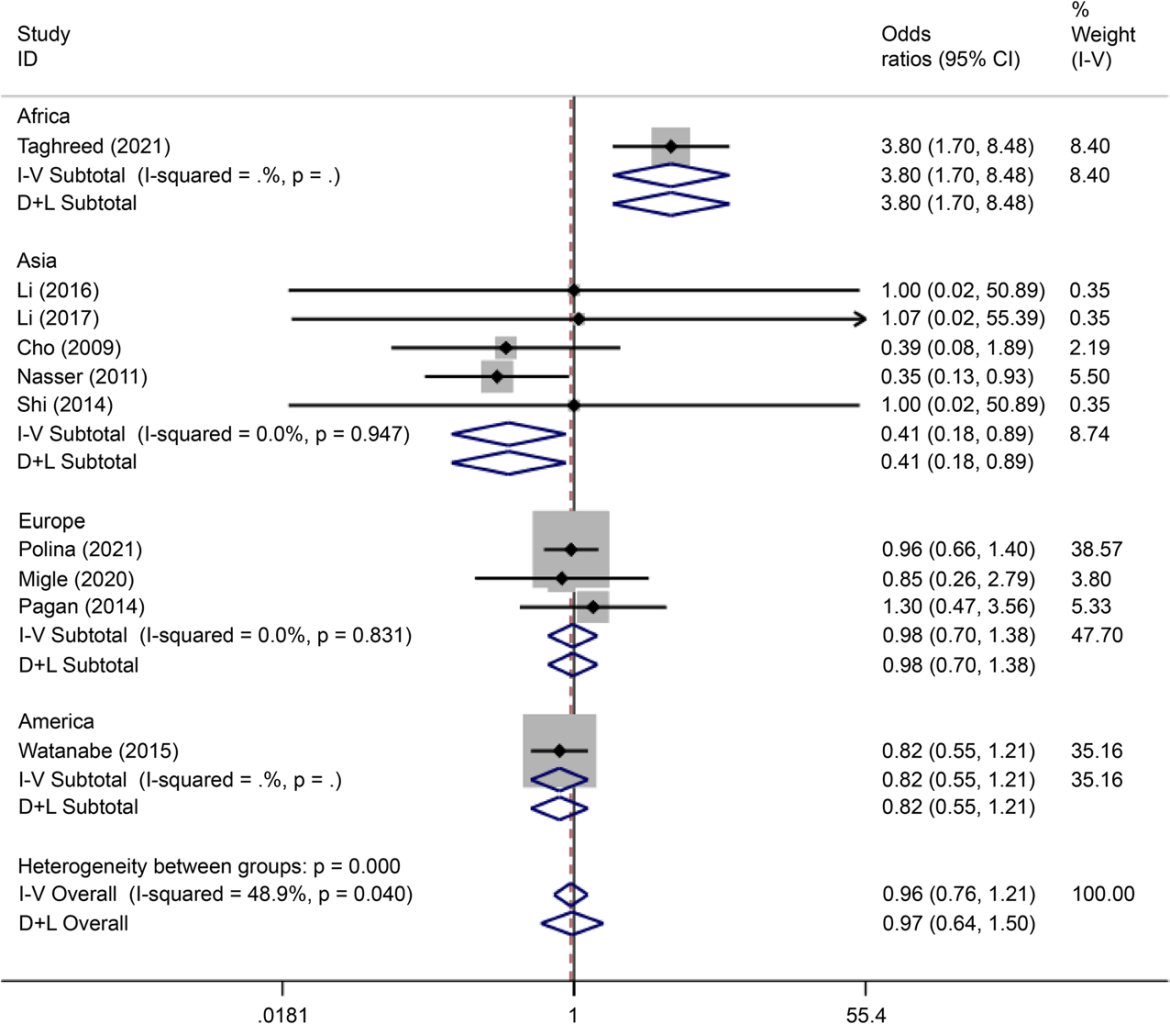
Forest plot of the relationship between GDM and rs 12,255,372 SNP under recessive model. GDM, gestational diabetes mellitus; SNP, single nucleotide polymorphisms. Black diamonds denote the odds ratio

There were 30 articles on rs12255372 (15 written in Chinese and 15 written in English) reporting data on 3,312 cases and 3,535 controls. The SNP was associated with the incidence of GDM (OR = 0.91, 95% CI: 0.52–1.30) in the African population based on analysis using all gene models apart from the over-dominant gene model. In the Asian population, this polymorphism was associated with the incidence of GDM (OR = 0.82, 95% CI: -1.50–0.13) only in the over-dominant gene model analysis (Figs. 8, 9, 10, 11, 12, 13).

#### Association between rs7901695 and GDM

**Fig. 14:**
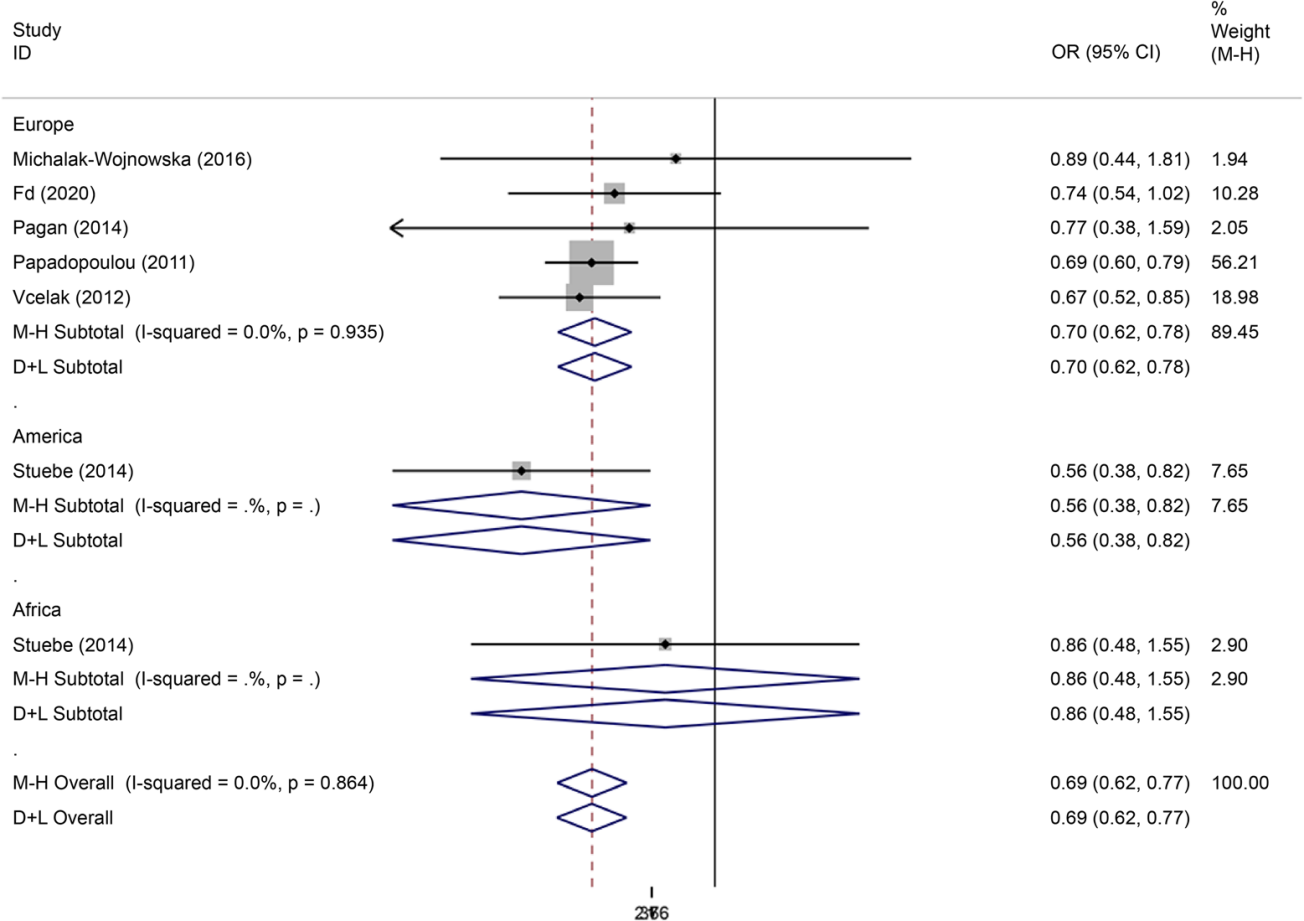
Forest plot of the relationship between GDM and rs 7,901,695 SNP under allelic model. GDM, gestational diabetes mellitus; SNP, single nucleotide polymorphisms. Black diamonds denote the odds ratio

**Fig. 15:**
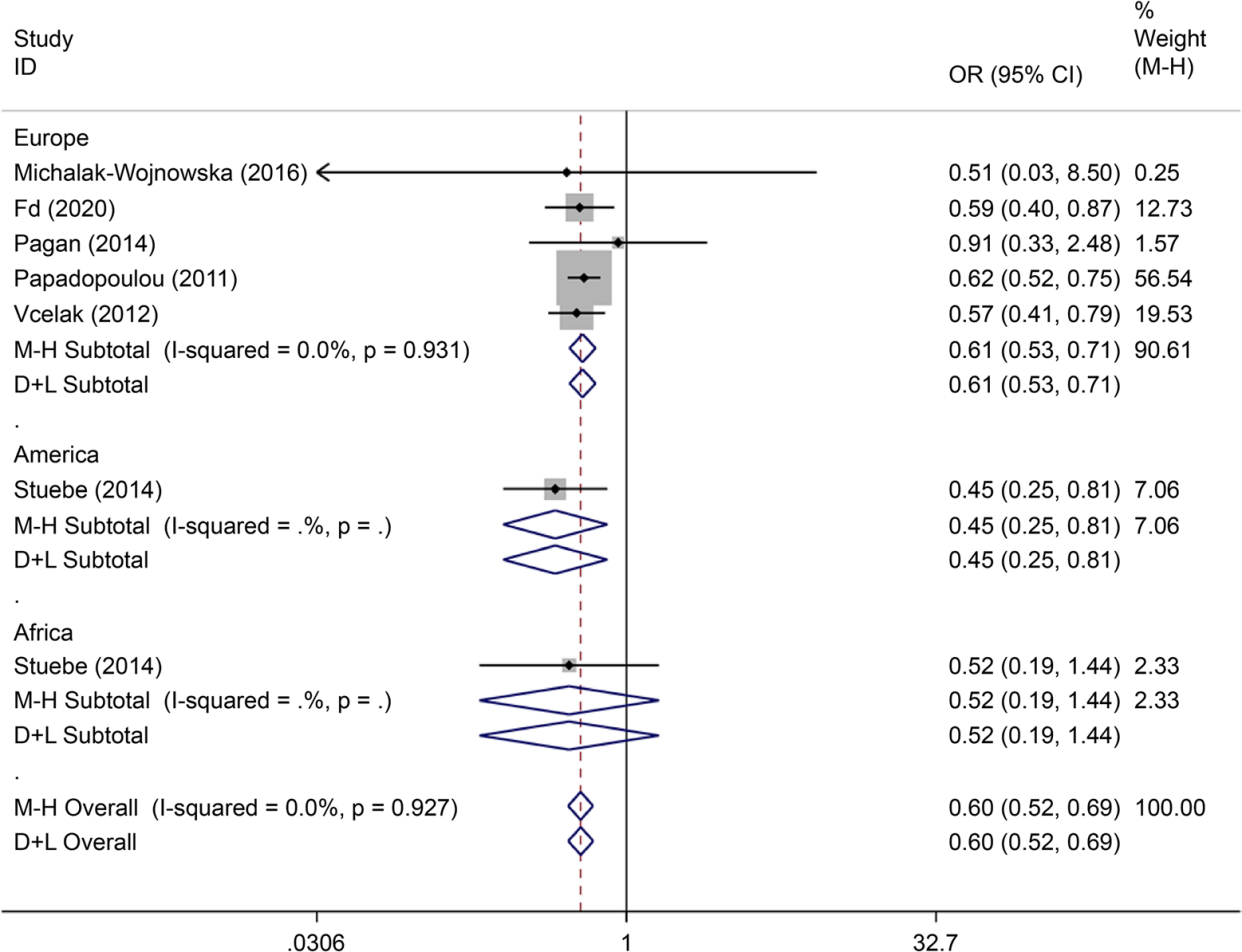
Forest plot of the relationship between GDM and rs 7,901,695 SNP under implicit model. GDM, gestational diabetes mellitus; SNP, single nucleotide polymorphisms. Black diamonds denote the odds ratio

**Fig. 16:**
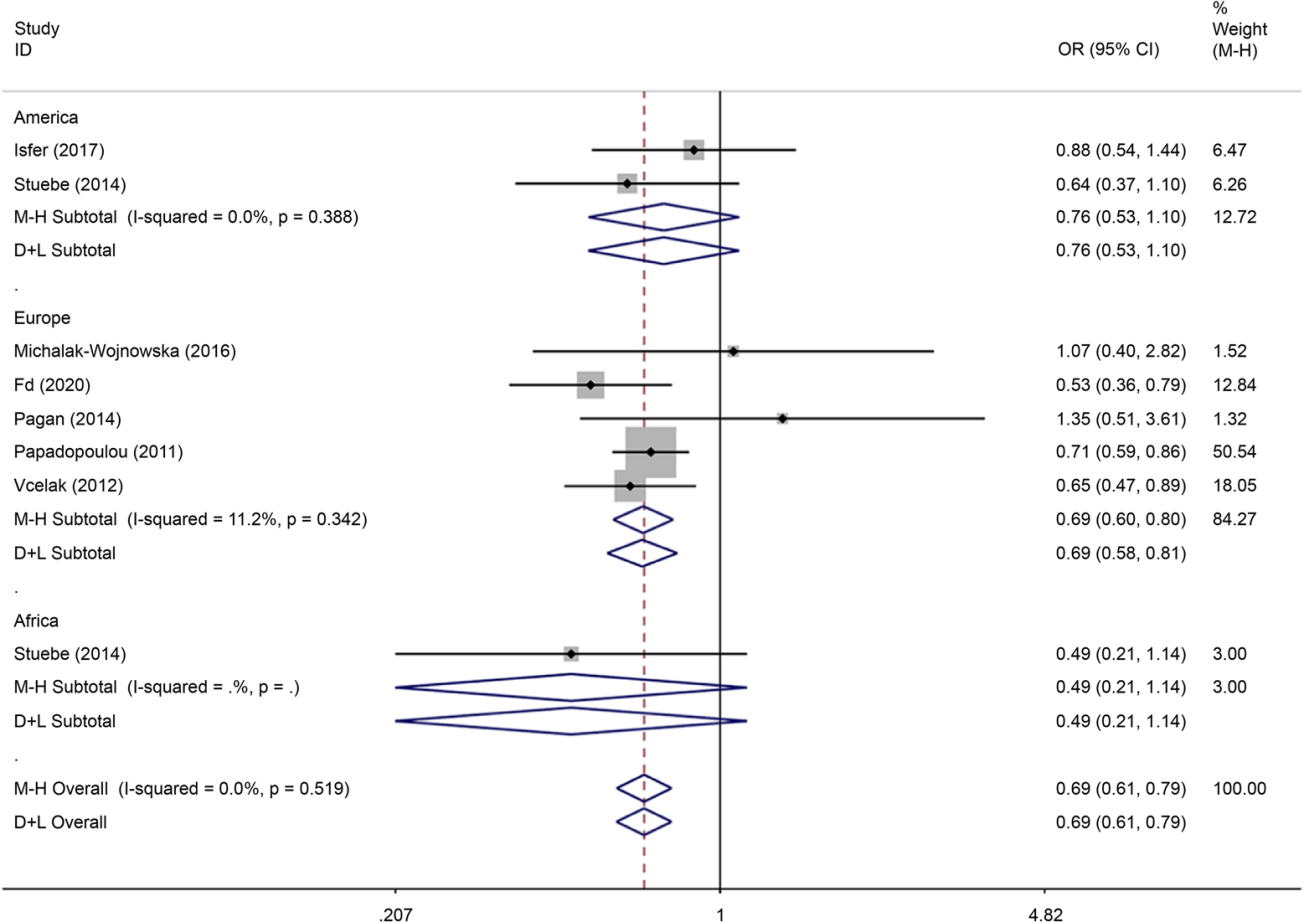
Forest plot of the relationship between GDM and rs 7,901,695 SNP under overdominant model. GDM, gestational diabetes mellitus; SNP, single nucleotide polymorphisms. Black diamonds denote the odds ratio

**Fig. 17:**
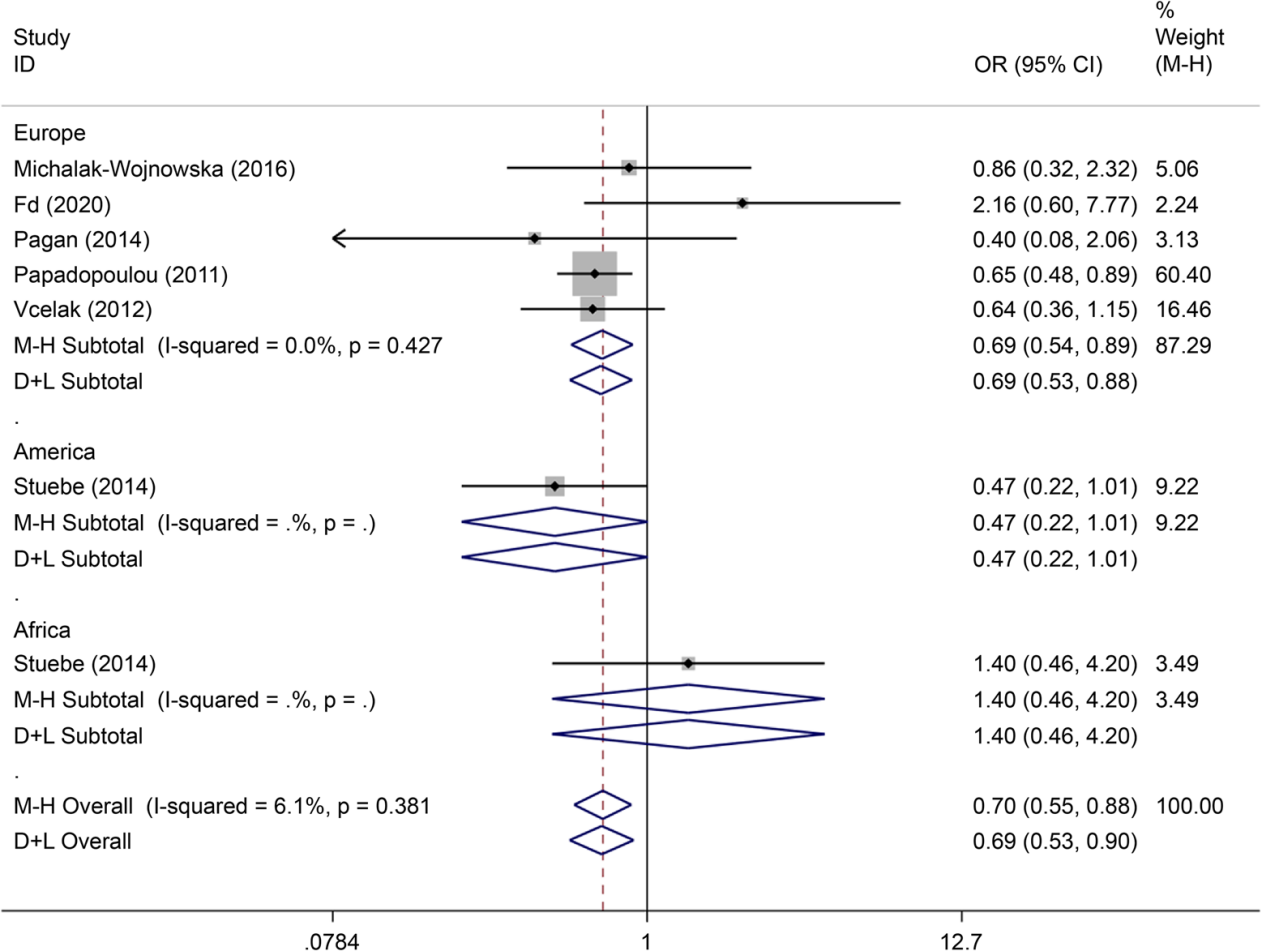
Forest plot of the relationship between GDM and rs 7,901,695 SNP under dominant model. GDM, gestational diabetes mellitus; SNP, single nucleotide polymorphisms. Black diamonds denote the odds ratio

**Fig. 18:**
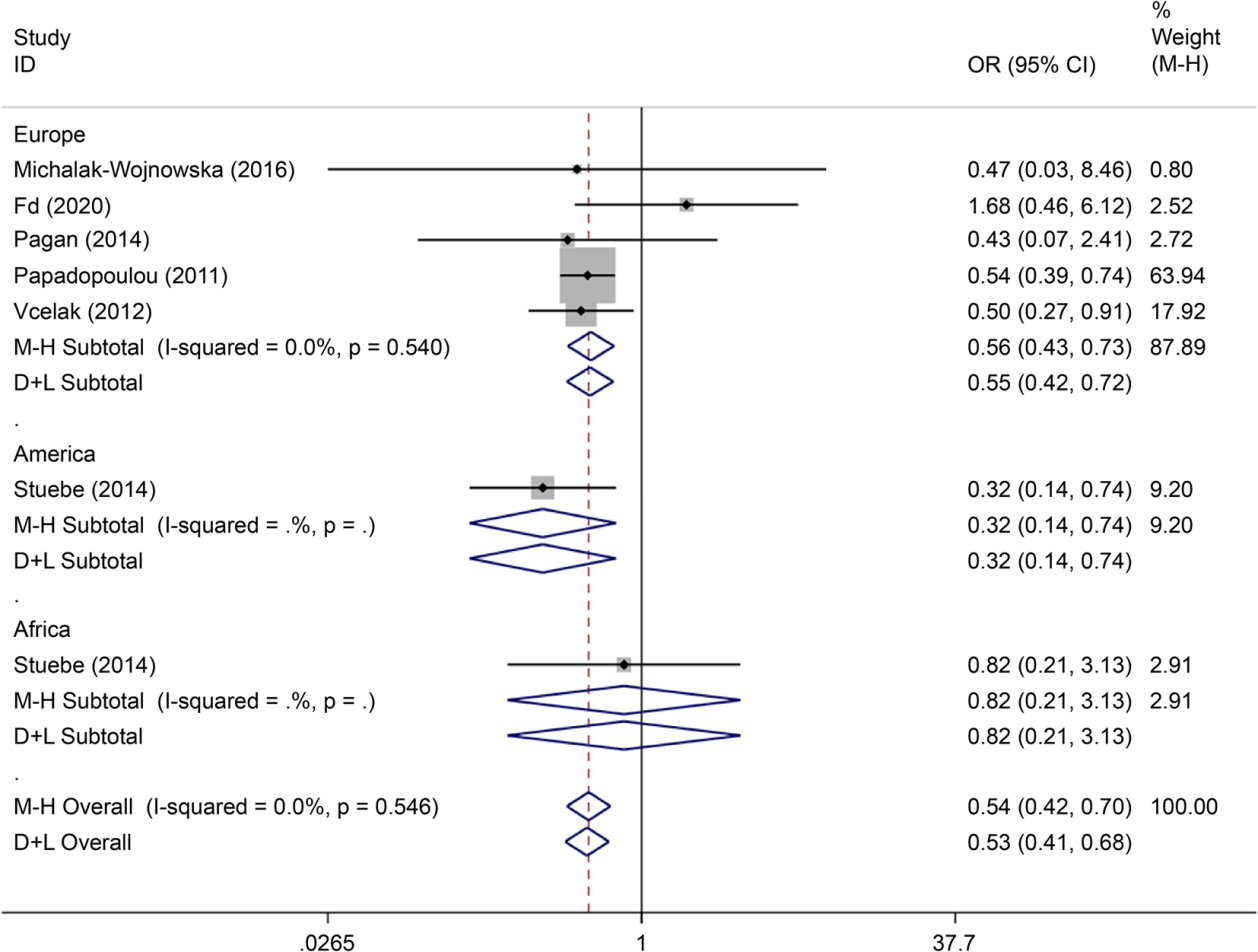
Forest plot of the relationship between GDM and rs 7,901,695 SNP under codominant model. GDM, gestational diabetes mellitus; SNP, single nucleotide polymorphisms. Black diamonds denote the odds ratio

There were eight articles on rs7901695 written in Chinese and English, reporting data on 1,515 cases and 3,157 controls. All five gene models showed that this SNP was associated with the onset of GDM (OR = 0.69, 95% CI: 0.61–0.79). All subgroup-based gene model analyses of this SNP showed associated incidence of GDM in the European population. Further, this SNP was associated with the incidence of GDM in the American population based on the allelic model (OR = 0.56, 95% CI: 0.38–0.82), the co-dominant genes model (R = 0.32, 95% CI: 0.14–0.74), and the recessive gene model (OR = 0.45, 95% CI: 0.25–0.81) (Figs. 14, 15, 16, 17, 18).

#### Association between rs290487 and GDM

**Fig. 19:**
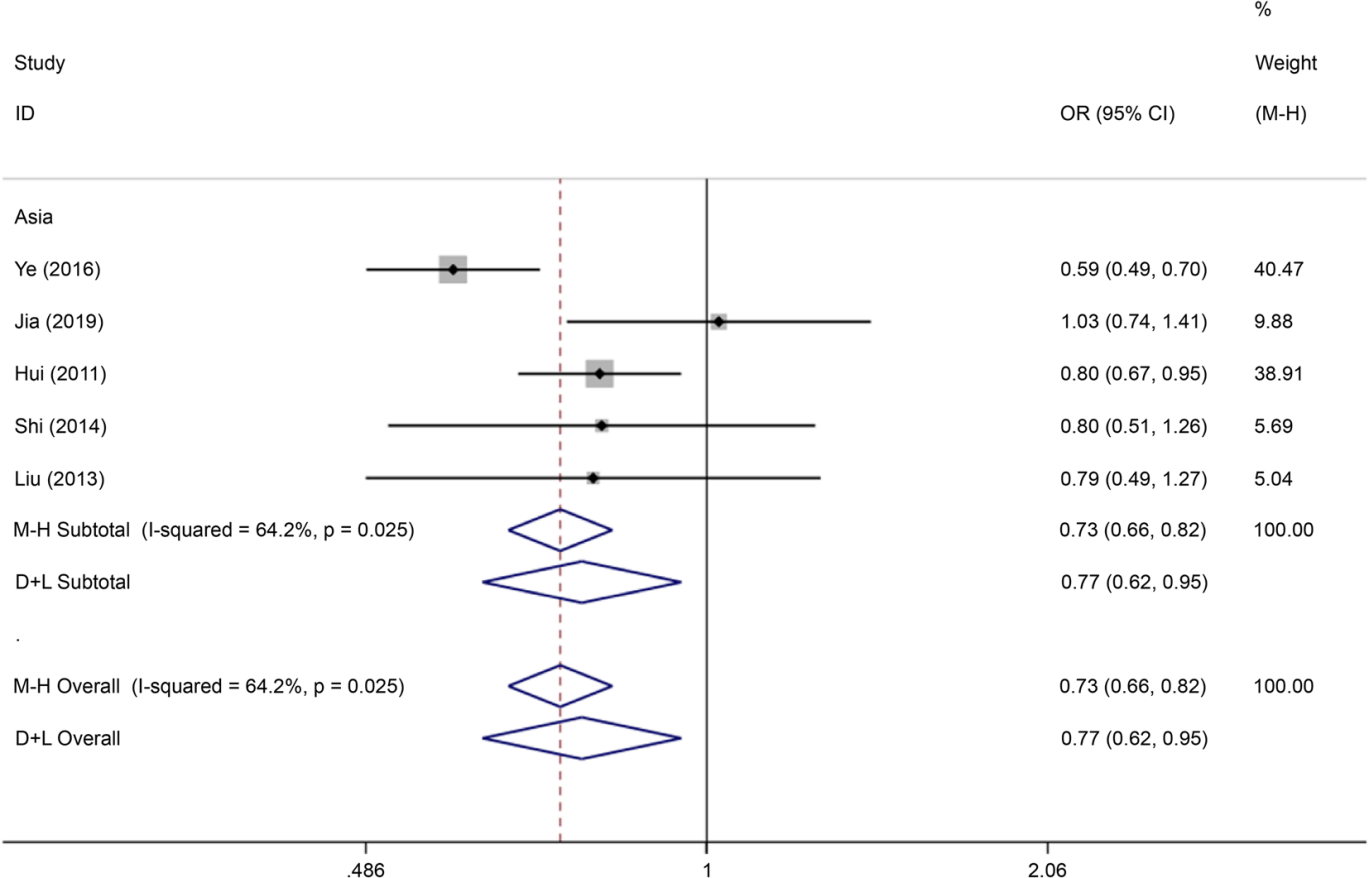
Forest plot of the relationship between GDM and rs 290,487 SNP under allelic model. GDM, gestational diabetes mellitus; SNP, single nucleotide polymorphisms. Black diamonds denote the odds ratio

**Fig. 20:**
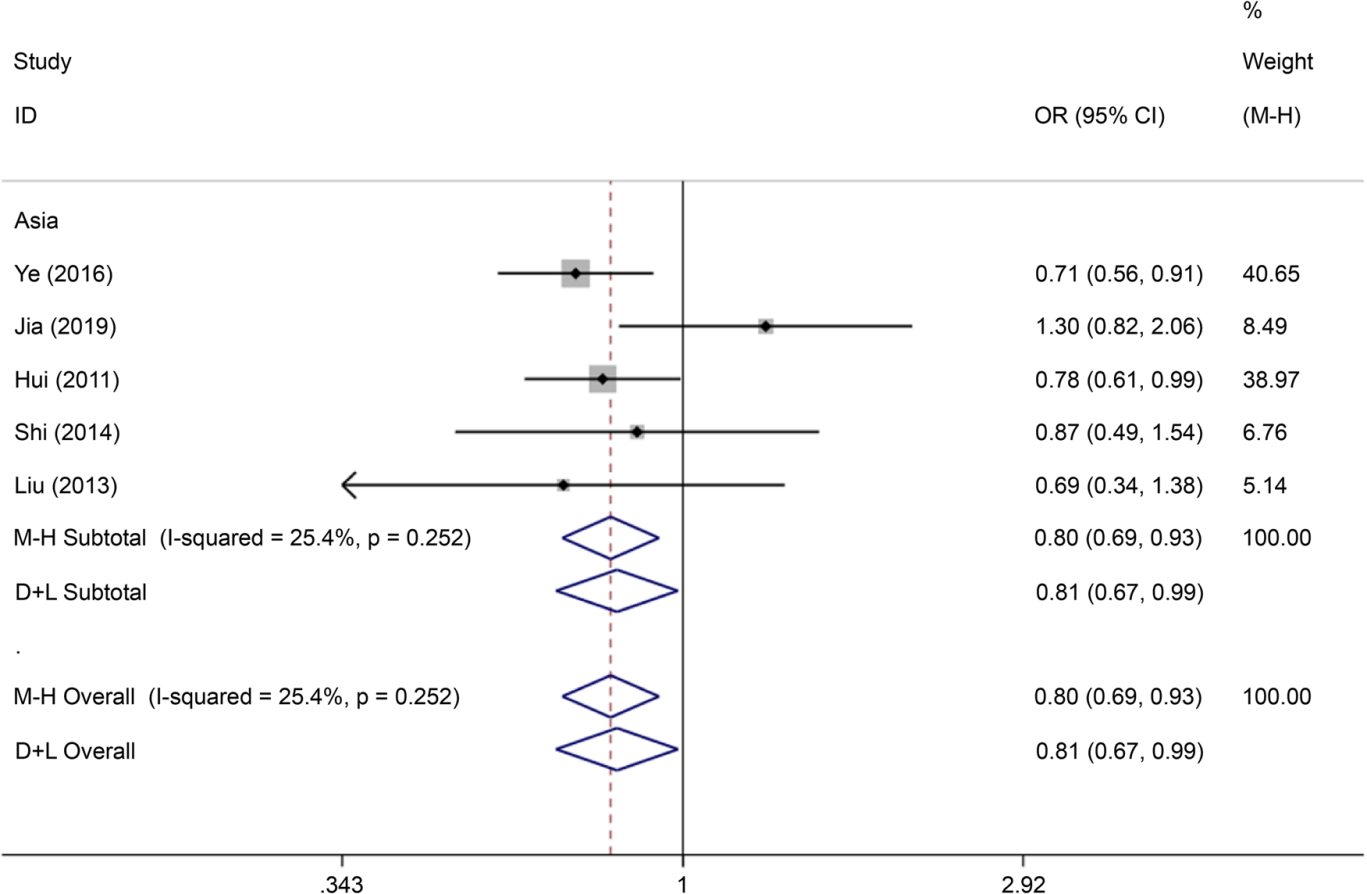
Forest plot of the relationship between GDM and rs 290,487 SNP under recessive model. GDM, gestational diabetes mellitus; SNP, single nucleotide polymorphisms. Black diamonds denote the odds ratio

There were five articles on rs290487 written in Chinese and English, reporting data on 1,361 cases and 1,361 controls. The allele model (OR = 0.73, 95% CI: 0.66–0.82) and recessive gene model (OR = 0.80, 95% CI: 0.69–0.93) demonstrated that this SNP was associated with the incidence of GDM (Figs. 19 and 20).

### Association between rs2975760 and GDM

**Fig. 21:**
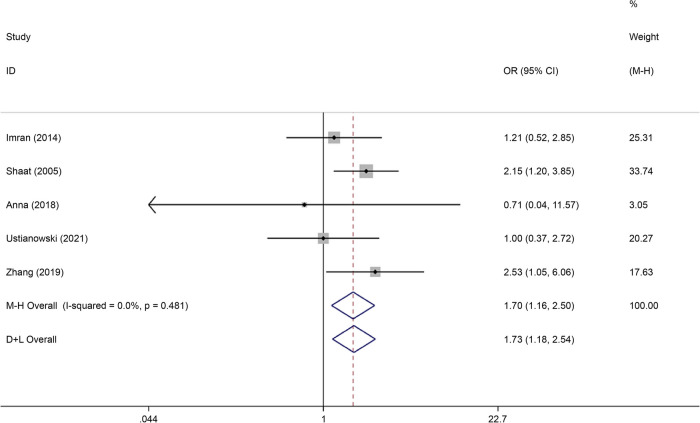
Forest plot of the relationship between GDM and rs 2,975,760 SNP under recessive model. GDM, gestational diabetes mellitus; SNP, single nucleotide polymorphisms. Black diamonds denote the odds ratio

Rs2975760 is the most studied SNP in *CAPN10*. A total of five studies on rs2975760, reporting data on 887 cases and 1,913 controls, were included in this analysis. Recessive gene model analysis demonstrated that this mutation was associated with the onset of GDM (OR = 1.70, 95% CI: 1.16–2.50) (Fig. 21).

## Discussion

In recent years, despite the large number of studies exploring the correlation between SNPs and GDM, there is a notable scarcity of high-quality meta-analyses within this domain.

Developments in molecular technologies have led to increased efforts to identify the genes associated with susceptibility to GDM as well as to develop molecular-based strategies for preventing and treating the disease [51]. The underlying mechanism of action of *TCF7L2* and *CAPN10* in the pathogenesis of GDM remains unclear [52], although *TCF7L2* and *CAPN10* may directly affect the function of pancreatic islet β-cells [53], resulting in decreased secretion of insulin and glucagon-like peptides, and subsequently leading to increased production of endogenous glucose [54]. Some studies have suggested that *TCF7L2*, an important gene associated with the pathogenesis of type 2 diabetes mellitus (T2DM), may interfere with GLP1, reduce the expression of GLP1R and glucose-dependent insulinotropic polypeptide/gastrointestinal inhibitory peptide (GIP) receptor (GIP-R), and inhibit β cell function by stimulating insulin secretion (in conjunction with GIP). *TCF7L2* may also lead to abnormal insulin conversion and activation of the Wnt signaling pathway, leading to T2DM [55, 56].

GDM has a similar pathogenesis to T2DM [57], and some studies have found that the two may also share genetic characteristics [58]. *TCF7L2* polymorphisms are also associated with susceptibility to GDM [59]. Studies in Sweden, Poland, and other European countries have suggested that the *TCF7L2* SNPs rs7903146 ​​and rs12255372 are associated with GDM pathogenesis [57]. Similar findings have been reported in Asian countries such as South Korea and China [43]. However, reports on the association between rs7901695 and rs290487 and GDM are still inconclusive, similar to the results of the subgroup analyses conducted in this study. The *TCF7L2* ​​SNP rs7903146 is a risk factor for GDM in Asian, European, American, African, and Pacific people. Unlike in previous studies, the subgroup analysis in our study showed that rs12255372, rs7901695, and rs290487 have different associations with GDM in different populations. The rs7903146 SNP variant, identified as a risk factor for individuals America, Asia, and the Pacific, may exhibit a protective role in the African population. This indicates that morbidity was associated with the onset of GDM, and a specific variant may serve as a protective or risk factor, depending on the population. For a more in-depth analysis of the reasons, it is crucial to consider the concept of genetic variability across populations. Populations demonstrate genetic diversity due to historical, geographical, and demographic factors. Therefore, a genetic variant that appears to confer protection in one population may not necessarily have the same effect in another. The interplay between genetic makeup and environmental factors can result in varying phenotypic expressions, making it essential to account for population-specific nuances in genetic studies [58]. Furthermore, the multifactorial nature of many health conditions necessitates a nuanced understanding of the role of specific variants. A variant might act as a protective factor in the presence of certain environmental conditions or in combination with other genetic factors. Conversely, the same variant may manifest as a risk factor under different circumstances. This emphasizes the need for comprehensive analyses that consider gene–gene and gene-environment interactions to unravel the intricate relationships between genetics and health [59]. Additionally, the observed variability in the effects of genetic variants might be attributed to gene flow and evolutionary processes. Migration and historical events can lead to the dissemination of certain genetic variants in specific populations, influencing their prevalence and impact on health outcomes [43].

Compared with *TCF7L2, CAPN10* has only few studies analyzing its association with GDM. rs2975760 and rs5030952 have been associated with GDM pathogenesis [46, 48, 49]. However, only rs2975760 was associated with the onset of GDM in this study.

This study had some limitations. First, we only retrieved articles written in Chinese and English. Hence, we may have missed articles written in other languages. Additionally, the meta-analysis might be susceptible to publication bias, where studies with positive results are more likely to be published than those with null findings. This can lead to an overestimation of the true effect size. Addressing these limitations would strengthen the robustness and applicability of the meta-analysis, providing a more comprehensive and nuanced understanding of the relationship between *TCF7L2* and *CAPN10* gene polymorphisms and GDM across different geographical regions.

## Conclusions

We searched eight Chinese and English databases: Cochrane, Elton B. Stephens. Company (EBSCO), Embase, Scopus, Web of Science, China National Knowledge Infrastructure (CNKI), Wanfang, and China Science and Technology Journal Database and retrieved all relevant articles published between the inception of the database and July 2022. As such, we found that rs7903146, rs12255372, rs7901695, rs290487, and rs2975760 were associated with the incidence of GDM in different populations.

## Data Availability

All data generated or analysed during this study are included in this published article.

## Abbreviations

GDM: Gestational diabetes mellitus
CNKI: China National Knowledge Infrastructure
NOS: Newcastle Ottawa Scale
OR: Odds ratio
SNPs: Single nucleotide polymorphisms
Cl: Confidence interval
GIP: Gastrointestinal inhibitory peptide
GIP-R: Gastrointestinal inhibitory peptide receptor
T2DM: Type 2 diabetes mellitus

## References

[1] Zhang M, Zhou Y, Zhong J, Ding Y, Li L (2019). The adaptation of clinical nursing practice guideline for gestational diabetes mellitus. Chin J Nurs.

[2] de la Torre NG, Assaf-Balut C, Jiménez Varas I, Del Valle L, Durán A, Fuentes M (2019). Effectiveness of following Mediterranean diet recommendations in the real world in the incidence of gestational diabetes mellitus (GDM) and adverse maternal-foetal outcomes: a prospective, universal, interventional study with a single group. The St Carlos Study. Nutrients.

[3] Mistry SK, Das Gupta R, Alam S, Kaur K, Shamim AA, Puthussery S (2021). Gestational diabetes mellitus (GDM) and adverse pregnancy outcome in South Asia: a systematic review. Endocrinol Diabetes Metab.

[4] Hewage SS, Wu S, Neelakantan N, Yoong J (2020). Systematic review of effectiveness and cost-effectiveness of lifestyle interventions to improve clinical diabetes outcome measures in women with a history of GDM. Clin Nutr ESPEN.

[5] Catalano PM, McIntyre HD, Cruickshank JK, McCance DR, Dyer AR, Metzger BE (2012). The hyperglycemia and adverse pregnancy outcome study: associations of GDM and obesity with pregnancy outcomes. Diabetes Care.

[6] Weschenfelder F, Baum N, Lehmann T, Schleußner E, Groten T (2020). The relevance of fetal abdominal subcutaneous tissue recording in predicting perinatal outcome of GDM pregnancies: a retrospective study. J Clin Med.

[7] Popova PV, Klyushina AA, Vasilyeva LB, Tkachuk AS, Vasukova EA, Anopova AD (2021). Association of common genetic risk variants with gestational diabetes mellitus and their role in GDM prediction. Front Endocrinol (Lausanne).

[8] Lewandowska M (2021). Gestational diabetes mellitus (GDM) risk for declared family history of diabetes, in combination with BMI categories. Int J Environ Res Public Health.

[9] Zhou M, Peng L, Wang J, Cao R, Ou Z, Fang Y (2022). Cadmium exposure and the risk of GDM: evidence emerging from the systematic review and meta-analysis. Environ Sci Pollut Res Int.

[10] Fritsche L, Sarief M, Wagner R, Stefan N, Lehmann R, Häring HU (2018). Genetic variation in TCF7L2 rs7903146 and history of GDM negatively and independently impact on diabetes-associated metabolic traits. Diabetes Res Clin Pract.

[11] Hou Z, Li M, Cao Y (2017). TCF7L2, CAPN10 polymorphisms are associated with gestational diabetes mellitus (GDM) risks: a meta-analysis. Gynecol Endocrinol.

[12] Norris JM, Simpson BS, Ball R, Freeman A, Kirkham A, Parry MA (2021). A modified Newcastle-Ottawa scale for assessment of study quality in genetic urological research. Eur Urol.

[13] Santos-Bueso E (2021). [Wieger’s ligament, Egger’s line and Berger’s space]. J Fr Ophtalmol.

[14] Shalabi TA, Amr KS, Shaker MM (2021). Are single nucleotide polymorphisms rs7903146 and rs12255372 in transcription factor 7-like 2 gene associated with an increased risk for gestational diabetes mellitus in Egyptian women?. J Genet Eng Biotechnol.

[15] Michalak-Wojnowska M, Gorczyca-Siudak D, Gorczyca T, Mosiewicz B, Kwaśniewska A, Filip A (2016). Association between rs7901695 and rs7903146 polymorphisms of the TCF7l2 gene and gestational diabetes in the population of Southern Poland. Ginekol Pol.

[16] Francaite-Daugeliene M, Lesauskaite V, Tamosiunas A, Jasukaitiene A, Velickienė D (2021). Genetic variants of TCF7L2 gene and its coherence with metabolic parameters in Lithuanian (Kaunas District) women population with previously diagnosed gestational diabetes mellitus compared to general population. Diabetes Res Clin Pract.

[17] Freathy RM, Hayes MG, Urbanek M, Lowe LP, Lee H, Ackerman C (2010). Hyperglycemia and adverse pregnancy outcome (HAPO) study: common genetic variants in GCK and TCF7L2 are associated with fasting and postchallenge glucose levels in pregnancy and with the new consensus definition of gestational diabetes mellitus from the International Association of Diabetes and Pregnancy Study Groups. Diabetes.

[18] Lauenborg J, Grarup N, Damm P, Borch-Johnsen K, Jørgensen T, Pedersen O (2009). Common type 2 diabetes risk gene variants associate with gestational diabetes. J Clin Endocrinol Metab.

[19] Pagán A, Sabater-Molina M, Olza J, Prieto-Sánchez MT, Blanco-Carnero JE, Parrilla JJ (2014). A gene variant in the transcription factor 7-like 2 (TCF7L2) is associated with an increased risk of gestational diabetes mellitus. Eur J Obstet Gynecol Reprod Biol.

[20] Papadopoulou A, Lynch KF, Shaat N, Håkansson R, Ivarsson SA, Berntorp K (2011). Gestational diabetes mellitus is associated with TCF7L2 gene polymorphisms Independent of HLA-DQB1*0602 genotypes and islet cell autoantibodies. Diabet Med.

[21] Pappa KI, Gazouli M, Economou K, Daskalakis G, Anastasiou E, Anagnou NP (2011). Gestational diabetes mellitus shares polymorphisms of genes associated with insulin resistance and type 2 diabetes in the Greek population. Gynecol Endocrinol.

[22] Shaat N, Lernmark A, Karlsson E, Ivarsson S, Parikh H, Berntorp K (2007). A variant in the transcription factor 7-like 2 (TCF7L2) gene is associated with an increased risk of gestational diabetes mellitus. Diabetologia.

[23] Včelák J, Vejražková D, Vaňková M, Lukášová P, Bradnová O, Hálková T (2012). T2D risk haplotypes of the TCF7L2 gene in the Czech population sample: the association with free fatty acids composition. Physiol Res.

[24] de Melo SF, Frigeri HR, dos Santos-Weiss IC, Réa RR, de Souza EM, Alberton D (2015). Polymorphisms in FTO and TCF7L2 genes of Euro-Brazilian women with gestational diabetes. Clin Biochem.

[25] Huerta-Chagoya A, Vázquez-Cárdenas P, Moreno-Macías H, Tapia-Maruri L, Rodríguez-Guillén R, López-Vite E (2015). Genetic determinants for gestational diabetes mellitus and related metabolic traits in Mexican women. PLoS One.

[26] Reyes-López R, Pérez-Luque E, Malacara JM (2014). Metabolic, hormonal characteristics and genetic variants of TCF7L2 associated with development of gestational diabetes mellitus in Mexican women. Diabetes Metab Res Rev.

[27] Yadav SK, Rashmi, Tripathi KK, Singh R (2016). Association of TCF7L2 gene variant with T2DM, T1DM and gestational diabetes in the population of northeastern UP, India. Int J Diabetes Dev Ctries.

[28] Li Y. Study on the association between TCF7L2 gene polymorphisms and gestational diabetes mellitus. Fujian Medical University; 2016.

[29] Chen Yugang, Fengying Y, Tong J, Bo L, Lingyin K, Juying S. Association of MTHFR and TCF7L2 gene polymorphisms with susceptibility to gestational diabetes. Matern Child Health Care China. 2019;34:4728–30.

[30] Chen Gaoqin ZY, Guangying H, Yi S (2019). Correlation between TCF7L2 gene locus rs7903146 T/C single nucleotide polymorphisms and genetic susceptibility in pregnant women with gestational diabetes. Chin J Birth Health Hered.

[31] Aris NKM, Ismai NAM, Mahdy ZA, Ahmad S, Naim NM, Siraj HHH (2011). An analysis of targeted single nucleotide polymorphisms for the risk prediction of gestational diabetes mellitus in a cohort of Malaysian patients. Asia Pac J Mol Med.

[32] Cho YM, Kim TH, Lim S, Choi SH, Shin HD, Lee HK (2009). Type 2 diabetes-associated genetic variants discovered in the recent genome-wide association studies are related to gestational diabetes mellitus in the Korean population. Diabetologia.

[33] Kan L, Lian A-F, Fei F-Y, Tan Y-J, Fei Y (2014). Study of the association in transcription factor 7-like2 (TCF7L2) gene polymorphism with gestational diabetes mellitus. Chin J Birth Health Hered.

[34] Rizk N, Rooshenas AA, Fouladi E, Rooshenas FA, Al-Ali K, Al-Khinji M et al. The associations of transcription factor 7-like 2 [TCF7L2] gene with gestational diabetes mellitus in State of Qatar. Diabetes. 2011;2011(Suppl 1).

[35] Zhou KC, Liu HW, Wang C, Fu YJ, Jin F (2014). Association of single nucleotide polymorphism of transcription factor 7 like 2 gene with gestational diabetes mellitus. Chin J Birth Health Hered.

[36] Thomas N, Mahesh DM, Chapla A, Paul J, Shwetha N, Christina F (2014). Does TCF7L2 polymorphisms increase the risk of gestational diabetes mellitus in South Indian population?. Endocr Abstracts.

[37] Zhang X (2015). Relationship between rs7903146-T/C polymorphism of TCFTL2 gene and gestational diabetes mellitus. J Chin Phys.

[38] Klein K, Haslinger P, Bancher-Todesca D, Leipold H, Knöfler M, Handisurya A (2012). Transcription factor 7-like 2 gene polymorphisms and gestational diabetes mellitus. J Matern Fetal Neonatal Med.

[39] Anghebem-Oliveira MI, Martins BR, Alberton D, Ramos EAS, Picheth G, Rego FGM (2017). Type 2 diabetes-associated genetic variants of FTO, LEPR, PPARg, and TCF7L2 in gestational diabetes in a Brazilian population. Arch Endocrinol Metab.

[40] Stuebe AM, Wise A, Nguyen T, Herring A, North KE, Siega-Riz AM (2014). Maternal genotype and gestational diabetes. Am J Perinatol.

[41] Shi X, Cai Q, Zou M, Shen Y (2014). Correlation between TCF7L2 gene polymorphism and genetic susceptibility in women with gestational diabetes mellitus. Zhonghua Fu Chan Ke Za Zhi.

[42] Watanabe RM, Allayee H, Xiang AH, Trigo E, Hartiala J, Lawrence JM (2007). Transcription factor 7-like 2 (TCF7L2) is associated with gestational diabetes mellitus and interacts with adiposity to alter insulin secretion in Mexican Americans. Diabetes.

[43] Ye D, Fei Y, Ling Q, Xu W, Zhang Z, Shu J (2016). Polymorphisms in TCF7L2 gene are associated with gestational diabetes mellitus in Chinese Han population. Sci Rep.

[44] Hui Y-C, Fan P, Wei L, Min N, Lihong Z, Hongding X (2011). Association of single nucleotide polymorphism of transcription factor 7 like 2 gene wim gestafional diabetes mellitus. Chin J Endocrinol Metab.

[45] Cui J, Xu X, Yin S, Chen F, Li P, Song C (2016). Meta-analysis of the association between four CAPN10 gene variants and gestational diabetes mellitus. Arch Gynecol Obstet.

[46] Shaat N, Ekelund M, Lernmark A, Ivarsson S, Almgren P, Berntorp K (2005). Association of the E23K polymorphism in the KCNJ11 gene with gestational diabetes mellitus. Diabetologia.

[47] Castro-Martínez AG, Sánchez-Corona J, Vázquez-Vargas AP, García-Zapién AG, López-Quintero A, Villalpando-Velazco HJ (2018). Association analysis of calpain 10 gene variants/haplotypes with gestational diabetes mellitus among Mexican women. Cell Mol Biol (Noisy-le-grand).

[48] Ustianowski P, Malinowski D, Kopytko P, Czerewaty M, Tarnowski M, Dziedziejko V (2021). ADCY5, CAPN10 and JAZF1 gene polymorphisms and placental expression in women with gestational diabetes. Life (Basel).

[49] Zhang X, Shi C, Wei L, Sun F, Ji L (2019). The association between the rs2975760 and rs3792267 single nucleotide polymorphisms of calpain 10 (CAPN10) and gestational diabetes mellitus. Med Sci Monit.

[50] Wojcik M, Zieleniak A, Zurawska-Klis M, Cypryk K, Wozniak LA (2016). Increased expression of immune-related genes in leukocytes of patients with diagnosed gestational diabetes mellitus (GDM). Exp Biol Med (Maywood).

[51] Niu S, Yu K, Wang W, Tan X, Xin H, Wu M. The expression and clinical value of miR-221 and miR-320 in the plasma of women with gestational diabetes mellitus. Clin Lab. 2022;68. 10.7754/Clin.Lab.2021.210927 35975537

[52] Liu K, Chen Y, Tong J, Yin A, Wu L, Niu J (2022). Association of maternal obesity with preterm birth phenotype and mediation effects of gestational diabetes mellitus and preeclampsia: a prospective cohort study. BMC Pregnancy Childbirth.

[53] Fritsche L, Heni M, Eckstein SS, Hummel J, Schürmann A, Häring HU (2022). Incretin hypersecretion in gestational diabetes mellitus. J Clin Endocrinol Metab.

[54] Biesecker LG (2018). Genomic screening for monogenic forms of diabetes. BMC Med.

[55] Ding W, Xu L, Zhang L, Han Z, Jiang Q, Wang Z (2018). Meta-analysis of association between TCF7L2 polymorphism rs7903146 and type 2 diabetes mellitus. BMC Med Genet.

[56] Karasek D, Krystynik O, Goldmannova D, Cibickova L, Schovanek J (2020). Circulating levels of selected adipokines in women with gestational diabetes and type 2 diabetes. J Appl Biomed.

[57] Zawiejska A, Wender-Ozegowska E, Bogacz A, Iciek R, Mikolajczak P, Brazert J (2018). An observational study of the risk of neonatal macrosomia, and early gestational diabetes associated with selected candidate genes for type 2 diabetes mellitus polymorphisms in women with gestational diabetes mellitus. Ginekol Pol.

[58] Lin P-C, Lin W-T, Yeh Y-H, Wung S-F (2016). Transcription factor 7-Like 2 (TCF7L2) rs7903146 polymorphism as a risk factor for gestational diabetes mellitus: a meta-analysis. PLoS One.

[59] Chang S, Wang Z, Wu L, Lu X, Shangguan S, Xin Y (2017). Association between TCF7L2 polymorphisms and gestational diabetes mellitus: a meta-analysis. J Diabetes Investig.

